# Application of a SSR‐GBS marker system on investigation of European Hedgehog species and their hybrid zone dynamics

**DOI:** 10.1002/ece3.4960

**Published:** 2019-02-14

**Authors:** Manuel Curto, Silvia Winter, Anna Seiter, Lukas Schmid, Klaus Scheicher, Leon M. F. Barthel, Jürgen Plass, Harald Meimberg

**Affiliations:** ^1^ Institute for Integrative Nature Conservation Research University of Natural Resources and Life Sciences (BOKU) Vienna Austria; ^2^ Division of Plant Protection University of Natural Resources and Life Sciences (BOKU) Vienna Austria; ^3^ Institute of Mathematics University of Natural Resources and Life Sciences (BOKU) Vienna Austria; ^4^ Evolutionary Ecology Leibniz Institute for Zoo and Wildlife Research (IZW) Berlin Germany; ^5^ Biologiezentrum Linz Oberösterreich Landesmuseum Linz Austria

**Keywords:** European hedgehog, hybridzone, microsatellites, SSR‐GBS, white‐breasted hedgehog

## Abstract

By applying second‐generation sequencing technologies to microsatellite genotyping, sequence information is produced which can result in high‐resolution population genetics analysis populations and increased replicability between runs and laboratories. In the present study, we establish an approach to study the genetic structure patterns of two European hedgehog species *Erinaceaus europaeus* and *E. roumanicus*. These species are usually associated with human settlements and are good models to study anthropogenic impacts on the genetic diversity of wild populations. The short sequence repeats genotyping by sequence (SSR‐GBS) method presented uses amplicon sequences to determine genotypes for which allelic variants can be defined according to both length and single nucleotide polymorphisms (SNPs). To evaluate whether complete sequence information improved genetic structure definition, we compared this information with datasets based solely on length information. We identified a total of 42 markers which were successfully amplified in both species. Overall, genotyping based on complete sequence information resulted in a higher number of alleles, as well as greater genetic diversity and differentiation between species. Additionally, the structure patterns were slightly clearer with a division between both species and some potential hybrids. There was some degree of genetic structure within species, although only in *E. roumanicus* was this related to geographical distance. The statistically significant results obtained by SSR‐GBS demonstrate that it is superior to electrophoresis‐based methods for SSR genotyping. Moreover, the greater reproducibility and throughput with lower effort which can be obtained with SSR‐GBS and the possibility to include degraded DNA into the analysis, allow for continued relevance of SSR markers during the genomic era.

## 1 INTRODUCTION

Second‐generation sequencing technologies are revolutionizing not only genome‐wide analyses, but also genotyping approaches. Several genotyping by sequencing methods have been developed and refined to the point that large parts of the genome can be covered, RAD‐sequencing (Restriction Site associated DNA) being the most prominent example (Andrews, Good, Miller, Luikart, & Hohenloh et al., 2016). Additionally, NGS (next‐generation sequencing) technologies have a large potential for traditional microsatellite (simple sequence repeat, SSR) analysis (de Barba et al., 2017). Although RAD‐sequencing methods are becoming more widely adopted, they still require relatively high coverage per locus and thus high‐throughput sequencing (Hodel et al., 2016). With lower coverage, the amount of missing data increases, compromising population genetic analyses of the subsequent datasets (Arnold, Corbett‐Detig, Hartl, & Bomblies, 2013; Curto, Schachtler, Puppo, & Meimberg, 2018).

Here, we use the term genotyping by sequencing (GBS) in the context of Elshire et al. (2011) and Vartia et al. (2016), referring to the genotype determination via second‐generation sequencing data, Illumina being the most commonly used technology. At its most extreme, GBS is whole‐genome analysis applications such as the resequencing of population pools and individuals, as exemplified by the dense SNP genotyping in human population genetics (e.g., 1000 Genomes Project Consortium, 2010; Li & Durbin, 2011) and animal breeding (e.g., Rubin et al., 2010; Daetwyler et al., 2014). As for most systems a reference genome is unavailable, downsizing is required, thus allowing the investigation of only a subset of loci within the genome (Cronn et al., 2012). Examples of these reduced representation approaches are the following: RAD‐sequencing (Baird et al., 2008), exon capture (Lemmon, Emme, & Lemmon, 2012), and amplicon sequencing. This last approach is genome downsizing to the largest extent, as only unique regions of the genome, such as single nucleotide polymorphisms (SNPs), are targeted. These methods can be further modified to fit high‐throughput approaches, such as with the use of inversion probes or genotyping by the thousand approaches (Campbell, Harmon, & Narum, 2015; Hardenbol et al., 2003).

Amplicon sequencing has a special role in SSR analysis (de Barba et al., 2017; Farrell, Carlsson, & Carlsson, 2016; Vartia et al., 2016; Šarhanová et al., 2018), and microsatellite amplification is the method of choice for population genetics, due to the ability to recover multiple alleles per locus, resulting in a high statistical power with a low number of sequenced markers (Ellegren, 2004; Schlotterer, 2000). Despite the obvious advantages of whole‐genome sequencing approaches, genotyping‐specific loci is more cost‐effective and more easily implemented, which is also one of the arguments found in recent reviews for the use of microsatellites in place of RAD/GBS (Hodel et al., 2017, 2016). Second‐generation sequencing methods facilitate new, more powerful applications using microsatellite loci by increasing the data collected and the possibility to reach high statistical power by increasing the number of markers per sample and the number of alleles per marker (de Barba et al., 2017; Tibihika, Curto et al., 2018; Vartia et al., 2016). Using this method, it is now possible to recover the complete sequence composition of the locus, including the repeat motif and SNPs in the flanking region. This approach makes it possible to overcome homoplasy characteristics of microsatellites (Vartia et al., 2016; Šarhanová et al., 2018). In these cases, shared alleles resulting from homoplasy would have the same number of repetitions but different flanking regions. Additionally, the application of GBS to SSR markers (SSR‐GBS) leads to an improvement in the reproducibility of data produced by different laboratories. Although problems caused by stutter bands remain, limitations associated with machine‐specific biases, the need to use the same size standards or the “plus A peak” artifact do not apply to SSR‐GBS. For these reasons, SSR markers are one of the most promising and obvious choices for GBS applications, and SSR‐GBS has the potential to overcome some of the shortcomings associated with traditional microsatellite analysis when compared to RADs (Hodel et al., 2017, 2016).

The primary advantage of RAD‐seq is the high number of SNPs that can be detected across the genome with relatively low cost and without previous genomic information (Andolfatto et al., 2011; Smith et al., 2010; Sonah et al., 2013). The high number of loci recovered with RAD‐seq allows for the recovery of population genetic differentiation patterns (Schopen, Bovenhuis, Visker, & Van Arendonk, 2008). However, there are some limitations associated with RAD‐seq, such as the difficulty in detecting paralogs without a reference genome, the high amount of missing data, and biases caused by the use of restriction enzymes that influence heterozygosity estimates, especially when stringent data filtering is implemented (Hodel et al., 2017). Further, SSR markers’ costs and data collection efforts do not increase linearly as a function of sample size. This compares favorably to RAD‐seq when genotyping high numbers of individuals (in the order of thousands), or for short‐term projects (Hodel et al., 2016). With the lower costs of the SSR‐GBS approach, this advantage is expected to be even greater. In this respect, SSR‐GBS has similarities with the genotyping by the thousands approach (Campbell et al., 2015).

In this paper, we present the development of SSR markers and their application in multiplexed amplifications to measure genetic variation in two species of hedgehog: the European hedgehog (*Erinaceus europaeus*) and the northern white‐breasted hedgehog (*Erinaceus roumanicus*). Both species occur in Austria where their ranges form a contact zone. These ranges are classic examples of postglacial recolonization patterns and the formation of a secondary contact zone in response to this process (Hewitt, 1999; Santucci, Emerson, & Hewitt, 1998). It has been hypothesized that during the glacial periods, populations which found refuge in the Iberian and Italian peninsulas diverged from a common ancestor to *E. europaeus*, while those in the Balkans to *E. roumanicus *(Seddon, Santucci, Reeve, & Hewitt, 2001). Both species are closely related, but hybridization seems to only occur occasionally (Bogdanov, Bannikova, Pirusskii, & Formozov, 2009) and molecular markers support a clear genetic division between the two species, when they occur in sympatry (Bolfíková & Hulva, 2012). Thus, according to current knowledge, these species do not form a hybrid zone. However, all previous investigations of hybridization between these species performed thus far were based on a low number of markers. Both species seem to be generally present among human settlements (primarily in gardens/yards), but in the contact zone distribution of both species might be influenced by competition. Regardless, hedgehogs are species that are potentially impacted by fragmentation of their habitat by human infrastructures, roadways potentially being the most significant barriers for gene flow and migration (Huijser & Bergers, 2000; Orlowski & Nowak, 2004). These hedgehog species have a moderate genetic structure, and on a larger scale, they show an isolation by distance pattern that is likely a consequence of recolonization after the last glaciation period (Bolfíková et al., 2017; Seddon et al., 2001). However, it has been verified that on small spatial scales the isolation by distance pattern can be disturbed due to habitat fragmentation and anthropogenic barriers to gene flow (Becher and Griffiths 1998), hence the importance of studying the genetic variation of these species in restricted geographical scales (Braaker, Kormann, Bontadina, & Obrist, 2017).

Second‐generation sequencing technologies provide new opportunities, in particular in studies where several species are examined. By increasing the information provided by genetic markers, one can detect genetic structure at smaller geographical scales and may be able to detect residual signs of hybridization that would otherwise be undetected (Corander & Marttinen, 2006; Ryman et al., 2006). Traditionally, microsatellite markers used in cross‐species amplification could potentially lead to bias favoring the species from which the markers originated (Turini et al., 2014). Additionally, biases in variability are also possible, which stem from modification, interruption or shortening of the repeat (Callen et al., 1993; Varshney, Graner, & Sorrells, 2005). Therefore, in addition to mismatches at the primer site leading to an increase in null alleles, markers might show less variability when used in cross‐species amplification.

Taking advantage of the Illumina technology, we developed markers from both species and tested their ability to amplify cross‐species markers. We determined the effectiveness of marker multiplexing to facilitate data collection and tested genotyping with the Illumina, using both length and sequence information in an SSR‐GBS approach, with tissue as well as noninvasive sampling, and outlined the results of genetic structure. The dataset we present here will form the basis of comprehensive studies of hedgehog genetic diversity, as well as investigations of introgression and gene flow between populations of the same and different species. Phylogeographic implications are outlined.

## 2 MATERIAL AND METHODS

### 2.1 Sampling and DNA isolation

A total of 82 individuals were used in the current study, 41 were identified as *E. europaeus *and 41 as *E. roumanicus *(Supporting Information Table S1). While most individuals were sampled in Austria, some were collected in other locations: one in Berlin, two in southeast Germany (Bavaria) near the border with Austria, two in eastern Slovakia, five in southwestern Czech Republic, one in northwestern Croatia, one in Hungary, and one in Macedonia. Sampling in Austria was concentrated in the areas surrounding Linz (35). Within this area, we subdivided the samples into four sub‐regions: Southeast Linz (3), East Linz (5), Linz (13), and West Linz (14). Four samples were collected in the areas surrounding Vienna in the province of Lower Austria, three of them in the region east of the city and one west of the city. Six samples were from southeast Austria in the province of Burgenland, five of them collected east of the lake Neusiedlersee. Twenty‐four samples were collected by three animal shelters: seven in Bludenz (Vorarlberg) and in Innsbruck (Tirol) in western Austria and 10 in Klagenfurt (Carinthia) in southern Austria. According to information from the shelters, these individuals were found within 100 km radius of the shelter and within the same province. Shelter samples were collected using mouth swabs from live animals, with the remaining ones collected as tissue samples from road fatalities. Individual samples were collected by several institutions (Supporting Information Table S1): the Biologiezentrum Linz, the Natural History Museum in Vienna, Leibniz Institute for Zoo and Wildlife Research, and the animal shelters.

For DNA isolation of buccal swabs, the swabs were placed in 500 µl lysis buffer (2% SDS, 2% PVP‐40, 250 mM NaCl, 200 mM Tris‐HCl, 5 mM EDTA, pH 8) and 16.67 µl of proteinase K (10 mg/ml) and incubated for 2.5 hr at 56°C. They were then removed with clean tweezers and placed in a NucleoSpin filter columns and centrifuged for 1 min at 562 *g*. For DNA purification, 400 µl of the supernatant were mixed with 15 µl of MagSi‐DNA beads (size 300 nm, MagSi‐DNA beads from MagnaMedics) and 600 µl binding buffer (2 M GuHCl in 95% ethanol) and incubated at room temperature for 5 min. The supernatant was separated from the beads by placing samples on the magnetic separator SL‐MagSep96 (Steinbrenner, Germany) for one minute. The beads were washed twice with 600 µl of 80% ethanol. To remove excess ethanol, the beads were air‐dried at room temperature for 10 min. Two elutions were made with 20 and 25 µl preheated (65°C) elution buffer (10 nM Tris with a pH of 8), and the beads were mixed with elution buffer and incubated for 5 min at room temperature. Tissue samples were isolated by the same procedure, with the exception that the product of lysis required no filtration, and the DNA was eluted in 30 and 50 µl of elution buffer.

### 2.2 Marker development

Marker development was conducted using two low‐coverage MiSeq runs, where one individual each of *E. europaeus* and *E. roumanicus *were sequenced using shot‐gun genomic libraries without enrichment. The *E. roumanicus *sample was roadkill from Romania. The *E. europaeus *sample stems from a sample collected in the area of Berlin. Both runs produced 300 bp paired‐end reads using libraries prepared with an insert length of between 400 and 500 bp to allow for sequence overlap. Raw reads of both runs are available in GenBank's SRA repository with the accession number PRJNA495814. Low‐quality regions and adapter sequences were trimmed using Cutadapt v. 0.11.1 (Martin, 2011), and the resulting reads were merged using PEAR vers. 0.9.4 (Zhang, Kobert, Flouri, & Stamatakis, 2013). These merged reads were used as input for the SSR_pipeline's script SSR_search.py in order to determine which sequences contained SSR motifs (Miller, Knaus, Mullins, & Haig, 2013). The following steps of quality control were included: The sequence contained a minimum of 40 bp flanking both sides of the motif; a minimum of six repeats for tetra‐ and pentanucleotide; a minimum of eight repeats for trinucleotides; and 10 repeats for dinucleotides. The number of sequences generated in the size range (350–550 bp) was sufficient for extracting a large number of microsatellite motif‐containing sequences. Sequences containing interruptions of the motif and mononuclear stretches larger than six bp were manually excluded; however, for some motif types this step resulted in too low number of usable reads was not feasible, and in these cases some mononucleotide repeats were accepted.

Primers were constructed using Primer3 (Untergasser et al., 2012) as implemented in Geneious v. 8.1.8 (Kearse et al., 2012) as a batch job under manual control. We only retained primers which produced amplicons containing the complete microsatellite repetition motif in the first or last 300 bases. This allowed the merging of paired reads in 300 bp MiSeq runs. Primers were designed to be between 19 and 22 bp long, with an optimal melting temperature of 55ºC. These were elongated with a recognition sequence that corresponded to the Illumina adapter, the forward primer being elongated with part of the P5 motif (TCTTTCCCTACACGACGCTCTTCCGATCT) and the reverse with part of the P7 motif (CTGGAGTTCAGACGTGTGCTCTTCCGATCT). These recognition sequences are necessary for a second PCR where eight‐bp index information and the rest of the Illumina adapters are added (P5: AATGATACGGCGACCACCGAGATCTACAC [Index] ACACTCTTTCCCTACACGACG; and P7:CAAGCAGAAGACGGCATACGAGAT [Index] GTGACTGGAGTTCAGACGTGT). Adapters were designed according to the Truseq chemistry because our initial experiments predated the release of the Nextera Chemistry that Illumina recommends for amplicon sequencing. For new experiments using this approach, the Nextera adaptors should be used.

### 2.3 SSR‐GBS amplicon library preparation

Primers were first tested individually in 10 µl PCRs containing 5 µl of QIAGEN Multiplex PCR Master Mix (Qiagen, CA, USA), 4 µl of each primer (1 µM), and 1 µl of template/genomic DNA. PCR was conducted using the following temperature profile: 95ºC for 15 min; 30 cycles of 95ºC for 30 s, 55ºC for 1 min, and 72ºC for 1 min; and a final extension at 72ºC for 10 min. PCR results were visualized using agarose gel electrophoresis, and primers which amplified a fragment of the correct size were combined in several primer mixes.

For genotyping, three runs were performed using relevant samples. The first included two samples which were amplified using different multiplex approaches: singleplex, and multiplexes of 4 and multiplex of 10 primer pairs, with the 35 *E. roumanicus *primer pairs. The 10 primer pair multiplex PCR was able to recover all loci; therefore, this approach was applied for the following runs. These comprised the same mixes of the *E. roumanicus *primers as above and a single mix of all *E. europaeus* primers. Primer mix solutions for multiplex PCR were composed of a combination of 10 to 30 primer pairs, each primer having a final concentration of 1 µM (Supporting Information Table S2). Multiplex amplification was performed using a protocol adapted from Curto et al. (2013). PCRs contained 0.5 µl of primer mix, 1 µl of DNA, 5 µl of QIAGEN Multiplex PCR Master Mix and water to complete the final reaction volume of 10 µl. All amplifications were performed using the same temperature profile as the single PCRs. PCR products from different primer mixes were mixed in equal volumes for each sample. This was primarily done to save time and cost, and a comparison with earlier experiments, where only a few primers were kept in multiplex (around 10), did not show an obvious change in the rate of success (e.g., increased dropout of loci and alleles).

Before proceeding to the second PCR, unused primers and primer dimer constructs were removed from the first PCR. PCR clean‐up was performed using magnetic bead technology following the protocol from Agencourt AMPure XP PCR Purification with some slight modifications. Four microlitres of PCR product was mixed with 2.86 µl of AMPure XP beads (Beckman Coulter Inc., Bree, CA, USA) and incubated for 5 min at room temperature. Bound DNA beads were captured by an inverted magnetic bead extraction device, VP 407‐AM‐N (V&P Scientific, INC.) and washed twice in an 80% 200 µl ethanol solution for 45 s. Later, the beads were dried at room temperature for 5 min and eluted in 17 µl of elution buffer (65 ºC 10 mM Tris‐Hcl, pH 8.3).

For the second PCR, a unique combination of forward and reverse indexes was chosen, allowing unambiguous identification of each sample after the MiSeq run. The PCR was conducted in a total volume of 10 µl containing 2 µl of each primer (1 µM), 5 µl of QIAGEN Multiplex PCR Master Mix, and 1 µl of purified PCR product. The reaction was carried out, after an initial denaturation and activation at 95ºC for 15 min, using 10 cycles of 95ºC for 30 s, 58ºC for 60 s, and 72ºC for 60 s. The reaction was incubated at 72°C for 5 min as a final extension. The resulting product consisted of the following from 5’ to 3’: (a) P5 motif for flow cell hybridization, (b) index 1 consisting of 8 bp, (c) P5 sequencing primer, (d) specific forward primer, (e) target DNA for sequencing; specific reverse primer; (f) P7 sequencing primer, (g) index 2 consisting of 8 bp, and (h) P7 motif for flow cell hybridization. In total, 10 different Index 1 and 10 different Index 2 sequences were used, allowing 100 different libraries to be sequenced simultaneously. PCRs were visualized on a 1.8% agarose gel and then pooled in equal volumes. Measurement of the DNA concentration was not performed as the fluctuation in DNA content within one Multiplex reaction was higher than between two reactions; it was therefore assumed that a normalization would not change the overall performance.

The resulting pool was used as input for an Illumina MiSeq run to produce sequences used for a genotyping by sequencing procedure. The pool, ca. 100 µl, was purified with magnetic bead technology, as described above, to remove possible dimers prior to Illumina sequencing. The amplicon libraries were sequenced in three runs with a calculated yield between 7.5 and 30 K sequences per DNA sample over all markers assuming an average of 15 M reads from a MiSeq run. Thus, it was expected that between 250 and 1,000 sequences per locus per sample would be obtained.

### 2.4 Sequence data extraction

**Figure 1:**
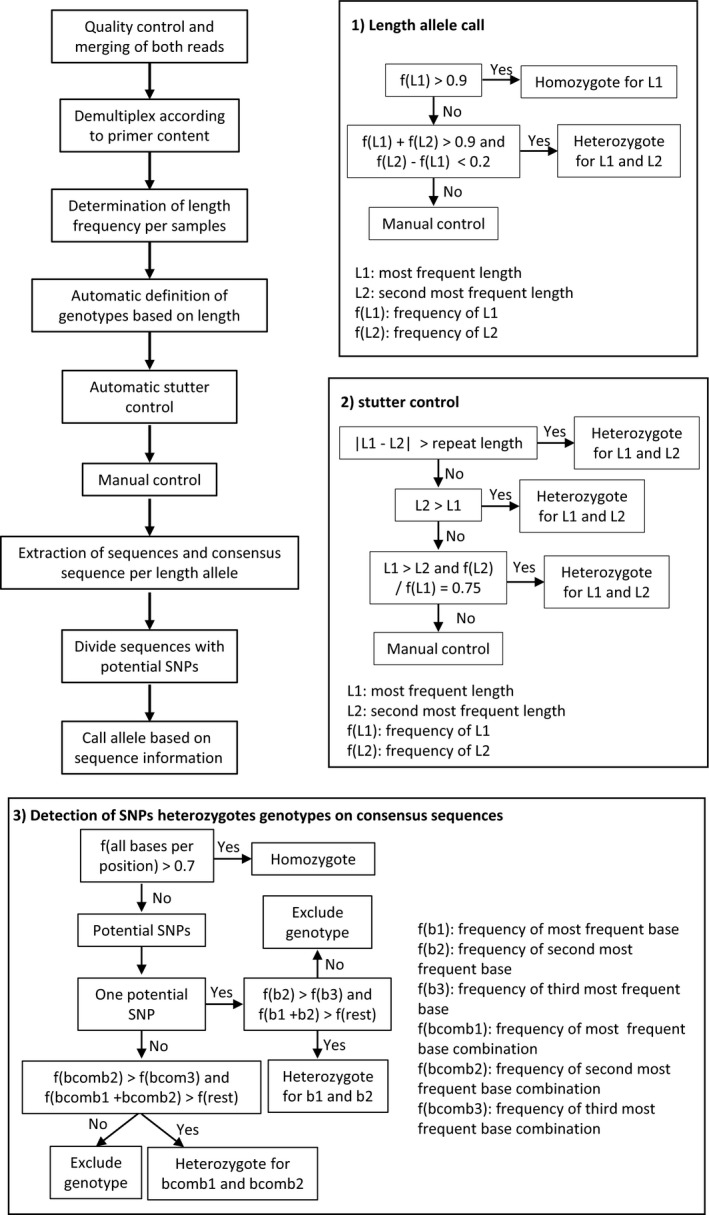
Summary of sequence analysis and genotyping approach. The top left panel shows the overview of the method. The right and bottom panels show decision trees concerning: allele call based on length (1), stutter control step (2), detection of SNP genotypes (3). L1 and L2 correspond to the two most frequent lengths found per sample and marker, while f(L1) and (L2) to their frequency. f(b1), f(b2), and f(b3) correspond to the frequencies of the most, second most and third most frequent nucleotides per position, respectively. f(bcomb1), f(bcomb2), and f(bcomb3) correspond, respectively, to the frequencies of the most, second most and third most frequent nucleotides combinations of two or more potential SNPs

The Illumina run was analyzed to determine sample genotypes in different steps (Figure 1). Extractions according to index combinations were automatically performed by the MiSeq machine, resulting in two fastq files containing all sequences per index, one for Read 1 and the other for Read 2. A combination of custom made scripts and third‐party programs was used for further processing of the samples, including quality control and trimming, merging of the paired reads, identification of primer sequences on both sides of sequences, and splitting the files according to primer sequences. Custom scripts were also used (Tibihika, Curto et al., 2018) and are available at github.com/mcurto/SSR‐GBS‐pipeline. First, paired reads were merged and quality controlled using the program PEAR. Reads were only merged if they overlapped for at least 10 bp with a *p*‐value below 0.01 for the highest observed expected alignment scores (OESs according to Zhang et al., 2013). Unmerged reads were not considered in further analyses. Merging was only possible because primers were designed to allow the complete microsatellite repetition motif to be sequenced by one of the paired reads. By doing so, it was also possible to assess the amplicon length. Previous to merging, low‐quality regions (Phred <20) were trimmed. In a second step, script 1 was used to identify the primer sequences on both sides of the merged reads and then sort them according to locus. According to our library preparation construct, the merged reads should start with the forward primer and end with the reverse primer sequence. All sequences not containing both primer motifs in the correct position were excluded. This step saved all sequences in one file by locus and sample. These files were used as input for subsequent genotyping analysis.

### 2.5 Allele definition

**Figure 2:**
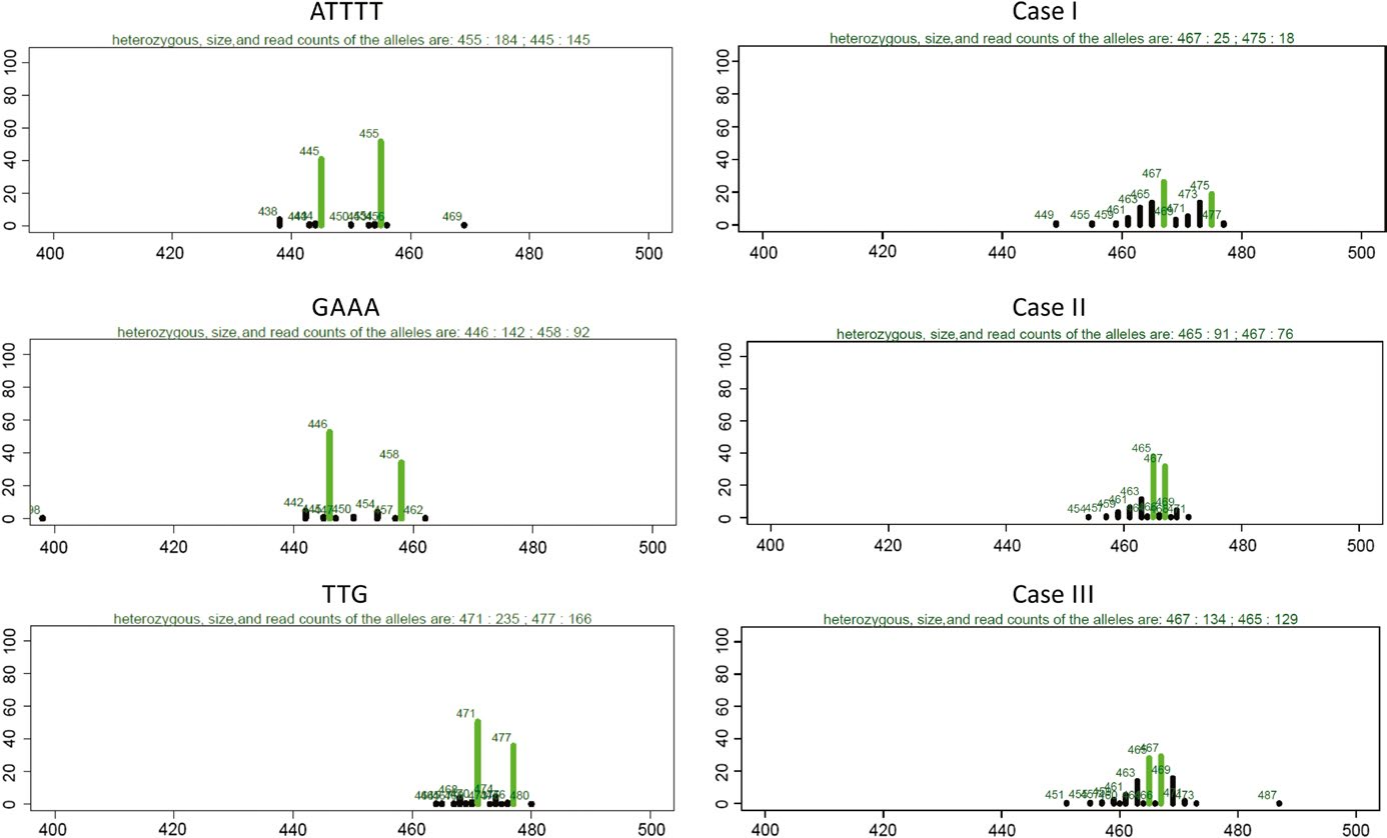
Number of reads per amplicon length. The left panel shows unambiguous heterozygote genotypes for tri‐,tetra‐,and pentanucleotide motifs. The right panel shows examples matching the three cases of the automatic stutter control: Case I, two alleles with a length difference above the repetition motif length; case II, two alleles with length difference equal to the motif length, whose the frequency of the shorter is higher than the longer one; case III, two alleles with length difference equal to the motif length, whose the frequency of the shortest allele is more than 75% of the longer one. Green bars correspond to amplicon lengths chosen as alleles by the genotyping method. Numbers above each bar indicate the allele length. The line above each graph indicates the chosen genotype and the corresponding number of reads supporting it

Alleles were defined based on the length of sequences and then on the occurrence of SNPs within each length class (Figure 1). With script 2 (Supporting Information), the sequence lengths occurring in one file and their corresponding counts were calculated and saved. Subsequently, all sequences with a length below a threshold (300 bp) were excluded from genotyping. Amplicons were constructed to be larger than 400 bp, so length of markers below this read length was likely artifacts and was excluded. Potential alleles were classified based on their length frequency using script 3 (Figure 1). Loci comprising one length with a frequency equal to or >90% of all reads were called homozygous for an allele characterized by the respective length. Genotypes were called heterozygous if the frequency of two lengths was >90% of reads and if the frequency of both lengths differed by no more than 20% (Figure 2). In a second step, the script 3 verified that the selected alleles were not the result of stutter. This was performed using the following three criteria (Figure 1): (a) the difference in length of the potential alleles is greater than one time the repeat motif length; (b) If condition one is not met, that is, if the two alleles differ by only one repeat, the allele of lower frequency must be longer than the one of higher frequency; (c) if condition two is not met, that is, if the two alleles differ in one repeat and the frequency of the shorter allele is lower than the frequency of the longer allele, then the shorter allele must have a frequency of 75% of the longer one. In Figure 2, we show one example of each case. The criteria were chosen in‐line with procedures used for allele calls based on chromatographic data. Programs (e.g., Genemapper, ABI as discussed in Johansson, Karlsson, & Gyllensten, 2003) frequently use the highest signal for allele call. In case of stutter bands in heterozygotes, the signal of the shorter allele and of a stutter band of the longer allele will be overlaid. This can lead to the shorter allele in a heterozygote having a stronger signal (or higher frequency in our case) than the longer allele. Our criteria take this into consideration and call a heterozygote if the stutter band pattern of a homozygote is interrupted (I), if one allele is potentially overlaid by stutter bands (II and III). After automated allele call, all data were plotted into histograms resulting in a graphic representation similar to traditional SSR chromatograms. This allowed for manual control of the allele call like standard for analysis using Genmapper or similar software (Meimberg et al., 2006). With this, our approach could be performed analogously to traditional fragment analysis. Generally, we were able to control for unspecific products. The typical stutter pattern of the homozygote genotypes and resulting from this the length frequency profile should look similar to a heterozygote genotype with overlaid stuttering. Only dinucleotide repeats required that a larger number of alleles be manually corrected. For penta‐, tetra‐, and trinucleotide repeats, the number of errors was very low and few corrections were necessary. All steps up until the geographical representation of frequencies and the table of genotypes according to length can be run automatically using the wrapping script *microsatPip*.

After manual control, sequences corresponding to the alleles based on length, were separated using the script 4 and condensed into one consensus sequence using the script 5. Frequencies of the most frequent nucleotide per position above 70% were considered homozygous and below 70% as potentially heterozygous. These heterozygous positions were indicated as ambiguous bases on the consensus sequence. For these cases, the consensus sequence was divided into two sequences based on the two most frequent nucleotides for that position using the script 6 (Figure 1). In the event that more than one SNP occurred in a sequence, these positions were considered as linked and the two most frequent nucleotide combinations were selected. If more than two equal frequency nucleotide combinations were found, the SNPs were either called by hand or left as ambiguous positions. In case this sample was already heterozygous for allele length, only the most frequent SNP combination was chosen. This approach was adopted under the assumption that sequencing errors and PCR errors such as chimeric sequences are less frequent than the sequences stemming from real alleles. For allele calling using the complete sequence information, each unique sequence (allele) was given a number and, according to which sequence was present for each sample, a codominant matrix was created. This was done using script 7. For comparison, the same was done with sequence length information, which was obtained after correcting the matrix produced by script 3.

### 2.6 Population genetics analysis

Population genetic analyses were performed using the codominant matrix as input with different standard programs. The dataset was analyzed for marker variability and polymorphism information content, as well as for genetic structure patterns among samples.

Variability measures per markers and population, such as number of alleles (*N*_a_) and observed (*H*_O_) versus expected (*H*_E_) heterozygosity, were calculated in GenAlEx v. 6.5 (Peakall & Smouse, 2006). Polymorphism information content (PIC) was obtained with the program Cervus v. 3.0.7 (Kalinowski, Taper, & Marshall, 2007). For comparison between genotyping approaches (length vs. complete sequence information) and primer sets (*E. europaeus‐ or E. roumanicus*‐specific primers), we also calculated genetic distances among individuals. This consisted of the average number of differing alleles per locus between each pair of samples. This was done using pairwise distance matrices containing the total number of different alleles per sample calculated with GenalEx. To facilitate graphical visualization, genetic distances were converted into average number of different alleles per locus. Differences between genotyping methods and marker sets for all above‐mentioned statistics were tested using the *t* tests as implemented in R v. 3.5.1 (R Core Team, 2018).

To evaluate genetic structure between species and populations without assumptions of Hardy–Weinberg Equilibrium (HWE), absolute genetic distances between individuals were calculated and the resulting matrix was used in a principal coordinates analyses (PCoA) as it is implemented in GenAlEx. This analysis was performed first using the complete dataset and then using only individuals from each species. All genetic structure analyses were done using both length and sequence information to test if the additional SNP information contributed to a more detailed genetic diversity pattern.

Sample clustering was evaluated using STRUCTURE v. 2.3.4 (Hubisz, Falush, Stephens, & Pritchard, 2009). This was done for datasets consisting of all samples, only *E. europaeus* and only *E. roumanicus*. To evaluate if genetic structure was affected by the use of species‐specific markers, STRUCTURE analyses were performed using either markers specifically designed for *E. europaeus *or *E. roumanicus*. Both length‐ and sequence‐based genotyping was used for these analyses. STRUCTURE was run using 15 independent replicates for 500,000 generations after a burn‐in period of 100,000. The admixture model and the allele frequencies among samples were considered to be correlated. *K*‐values between 1 and 10 were tested, and the *K*‐value was evaluated through the Delta‐K method implemented in the online program Structure Harvester, available at http://taylor0.biology.ucla.edu/structureHarvester/ (Earl, 2012). Replicates per *K*‐value were summarized using the online pipeline Clumpak (Kopelman, Mayzel, Jakobsson, Rosenberg, & Mayrose, 2015) available at http://clumpak.tau.ac.il/. To evaluate possible isolation by distance, a Mantel test was performed in GenAlEx comparing geographical and genetic distance matrices among individuals using the data produced from sequence information.

## 3 RESULTS

### 3.1 Marker development

For marker development, the MiSeq runs resulted in 2,201,005 and 1,348,477 paired reads for *E. roumanicus *and *E. europaeus*, respectively. After quality control and merging, a total of 1,464,370 and 716,091 reads were available for microsatellite motif screening. In total, 70,704 and 8,677 microsatellite containing sequences passed our criteria for *E. roumanicus *and *E. europaeus*, respectively. From these, there were 32,466 dinucleotide, 9,966 trinucleotide, 26,249 tetranucleotide, and 2,023 pentanucleotide repeats for *E. roumanicus*. For *E. europaeus*, there were 4,175 dinucleotide,730 trinucleotide, 3,539 tetranucleotide, and 233 pentanucleotide repeats. In total, 37 primers were designed for *E. roumanicus* and 34 for *E. europaeus*. Of these, 12 failed in the initial amplification step. The remaining primers are listed in Supporting Information Table S2.

### 3.2 Sequence analysis and genotyping

The three runs resulted in a total of 196,165, 842,591 and 1,790,852 paired reads, respectively. After quality control, paired read merging and primer demultiplex, 4,232,682 reads remained for all three runs. For each marker, the number of sequences varied between 268 and 446,616 per marker and between 12,664 and 136,247 per sample. The marker with the lowest number of sequences was W25_TTA and the one with the highest was W31_GA. Only 10 markers were not retained after the multiplex step: E25_TAC, E6_AAT, E32_ATCT, W20_TAGA, W24_ATA, W25_TTA, W26_TAT, W27_ATA, W3_AAAGA, and W5_AAAAT. These markers were not considered further despite based on singleplex reaction tests, they would have been able to be measured in less complex multiplex reactions.

Even though most markers were able to be amplified in both species, variability in the species from which they were not derived (non‐target species) was lower for many markers (Supporting Information Table S4). In five markers, the motif was missing in the non‐target species, and in three additional markers, the motif was interrupted and was less variable. In a few cases, alleles were fixed. In only a single case was a marker derived from *E. roumanicus* fixed in *E. roumanicus* but variable in *E. europaeus*. We excluded markers that were unable to produce genotypes for most samples (missing data >50%). This resulted in a total of 42 markers for further analysis. When only one species was analyzed after excluding markers based on missing data, only 42 markers remained for *E. europaeus *and 41 for *E. roumanicus*. Samples stemming from mouth swabs and tissue material contained on average 31% and 16% missing data, respectively. This corresponded to significantly higher missing data for mouth swabs samples when compared to tissue samples.

### 3.3 Marker variability

**Figure 3:**
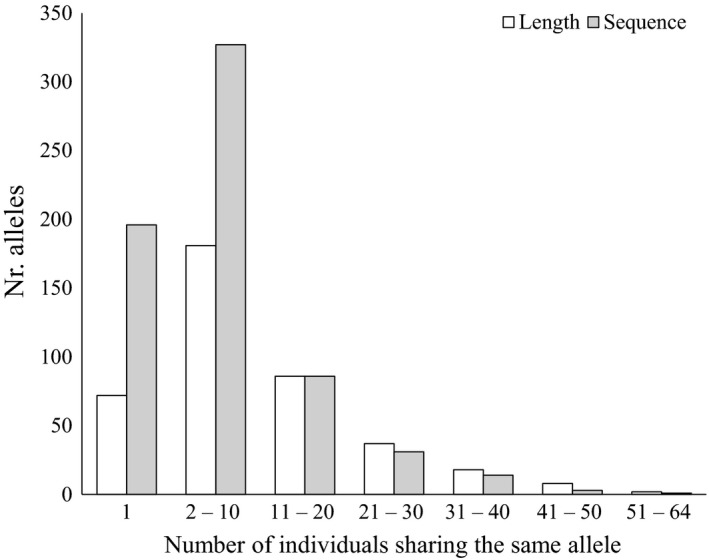
Number of alleles shared among individuals shown as the number of alleles (*y*‐axis) in dependence to the number individuals that share one allele (*x*‐axis). White and gray bars represent alleles called using sequence length information, respectively. The comparison includes the final 41 markers for all 82 individuals

Markers had between 1 and 23 alleles when only length polymorphisms were considered (Supporting Information Table S4; Table 1). When sequence information was included these numbers varied between 1 and 50 alleles. This corresponded to an increase in the number of singletons (72 for length and 196 for sequence information) and alleles shared among 2–10 individuals (Length = 181, Sequence = 327; Figure 3). There was no change in the number of alleles shared among 11 and 20 samples (86), while the allele call based on sequence information contributed to a decrease in the number of alleles shared among 21 or more individuals (Figure 3). One marker was monomorphic for the complete dataset including SNPs (E24_GCA) and two more were monomorphic in *E. roumanicus* (W15_ATAA) or *E. europaeus* (W13_TTTA). Considering length information and excluding the monomorphic markers, *H*_O_ varied between 0.09 and 1.00, *H*_E_ between 0.25 and 0.94, and PIC between 0.23 and 0.93. Including sequence information, *H*_O_ varied between 0.12 and 1.00, *H*_E_ between 0.49 and 0.97, and PIC between 0.46 and 0.96. The number of alleles within *E. europaeus*, excluding monomorphic markers, varied between 2 and 17 for length information and between 3 and 28 for sequence information. For the allele length dataset, *H*_O_ varied between 0 and 1.00, *H*_E_ between 0.05 and 0.89, and PIC between 0.05 and 0.87. When considering sequence information, the same values varied between 0.03 and 1, 0.07 and 0.94, and 0.11 and 0.94, respectively. For *E. roumanicus*, the number of alleles, excluding monomorphic markers, varied between 2 and 16 for length information and between 3 and 34 for sequence information. For the allele length dataset, *H*_O_ varied between 0 and 1.00, *H*_E_ between 0.07 and 0.92, and PIC between 0.07 and 0.91. When considering sequence information, the same values varied between 0 and 1, 0.11 and 0.96, and 0.07 and 0.93, respectively.

**Table 1:** Average, across used loci, of amplification success shown as percentage of missing data and average variability measures: *N*_a_—number of alleles, *H*_O_—observed heterozygosity, *H*_E_—expected heterozygosity, and PIC—polymorphism information content. Values in brackets correspond to minimum and maximum values. Values calculated based on sequence information are represented by the superscript “S” while the ones based on length information by “L”. Statistics were calculated based on different markers and samples sets

Statistics	Marker set	All samples	*E. europaeus*	*E. roumanicus*
% missing	All	15.48 (0–47.56)	14.75 (0–85.37)	16.2 (0–85.37)
	*E. europaeus*	15.39 (0–47.56)	18.01 (0–85.37)	12.76 (0–60.98)
	*E. roumanicus*	15.62 (0–45.12)	9.45 (0–51.22)	21.8 (0–85.37)
$N_{\text{a}}^{\text{L}}$	All	9.98 (2–23)	7.12 (2–17)	7.1 (2–16)
	*E. europaeus*	8.96 (3–19)	6.38 (2–13)	6.65 (3–13)
	*E. roumanicus*	11.63 (2–23)	8.31 (2–17)	7.81 (2–16)
$N_{\text{a}}^{\text{S}}$	All	16.83 (4–50)	10.45 (3–28)	10.38 (3–34)
	*E. europaeus*	15.58 (5–49)	9.54 (3–28)	10.15 (3–25)
	*E. roumanicus*	18.88 (4–50)	11.94 (3–23)	10.75 (4–34)
$H_{\text{o}}^{\text{L}}$	All	0.45 (0.09–1)	0.44 (0–1)	0.46 (0–1)
	*E. europaeus*	0.39 (0.09–0.97)	0.35 (0–0.97)	0.43 (0–0.98)
	*E. roumanicus*	0.55 (0.12–1)	0.59 (0.1–1)	0.51 (0.1–1)
$H_{\text{o}}^{\text{S}}$	All	0.52 (0.12–1)	0.51 (0.03–1)	0.51 (0–1)
	*E. europaeus*	0.47 (0.12–0.99)	0.44 (0.03–0.97)	0.49 (0–1)
	*E. roumanicus*	0.59 (0.12–1)	0.61 (0.1–1)	0.55 (0.12–1)
$H_{\text{E}}^{\text{L}}$	All	0.74 (0.25–0.94)	0.6 (0.05–0.89)	0.64 (0.09–0.92)
	*E. europaeus*	0.72 (0.47–0.92)	0.51 (0.05–0.89)	0.64 (0.09–0.9)
	*E. roumanicus*	0.76 (0.25–0.94)	0.74 (0.31–0.89)	0.65 (0.13–0.92)
$H_{\text{E}}^{\text{S}}$	All	0.81 (0.49–0.97)	0.68 (0.07–0.94)	0.71 (0.11–0.96)
	*E. europaeus*	0.8 (0.57–0.95)	0.62 (0.07–0.94)	0.71 (0.11–0.94)
	*E. roumanicus*	0.83 (0.49–0.97)	0.78 (0.41–0.94)	0.71 (0.16–0.96)
PIC^L^	All	0.7 (0.23–0.93)	0.56 (0.05–0.87)	0.6 (0.09–0.91)
	*E. europaeus*	0.68 (0.37–0.91)	0.48 (0.05–0.87)	0.59 (0.09–0.86)
	*E. roumanicus*	0.74 (0.23–0.93)	0.7 (0.29–0.87)	0.61 (0.12–0.91)
PIC^S^	All	0.78 (0.46–0.96)	0.67 (0.11–0.94)	0.64 (0.07–0.93)
	*E. europaeus*	0.77 (0.48–0.94)	0.67 (0.11–0.9)	0.58 (0.07–0.93)
	*E. roumanicus*	0.81 (0.46–0.96)	0.67 (0.16–0.94)	0.74 (0.38–0.92)

### 3.4 Comparison between genotyping approaches and species‐specific primers

**Figure 4:**
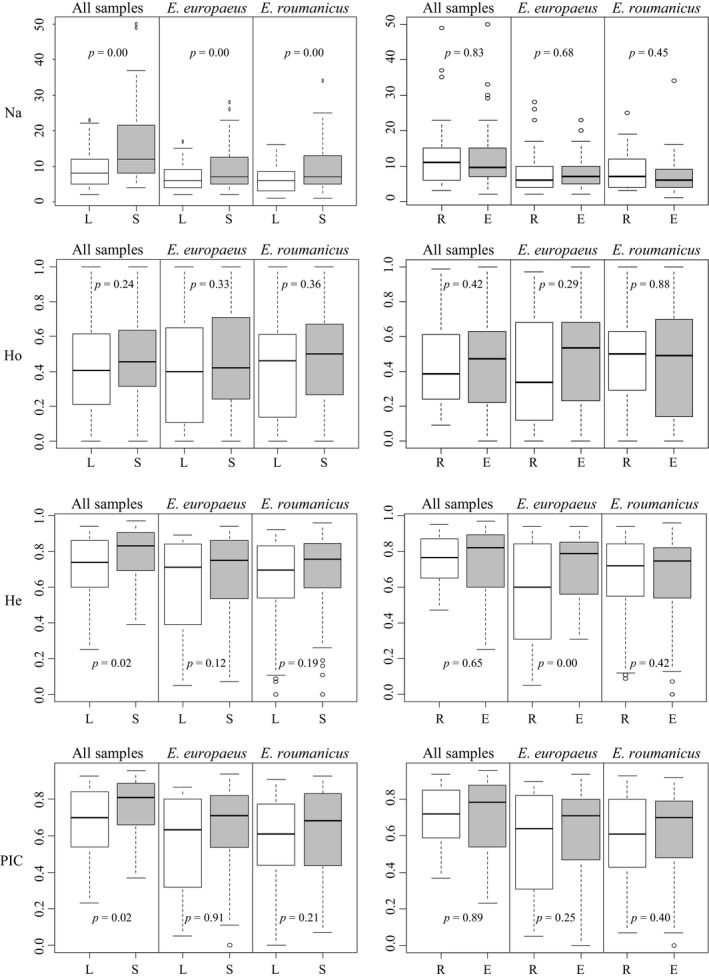
Boxplots describing variability and genetic diversity measurements per marker. Left panel using different allele calling approaches: sequence length (L) and sequence information (S). Right panel using different markers sets: *E. europaeus*‐specific primers (E) and *E. roumanicus* species primers (R). *p*‐Values correspond to *t* tests comparing differences in averages between genotyping methods and markers sets

**Figure 5:**
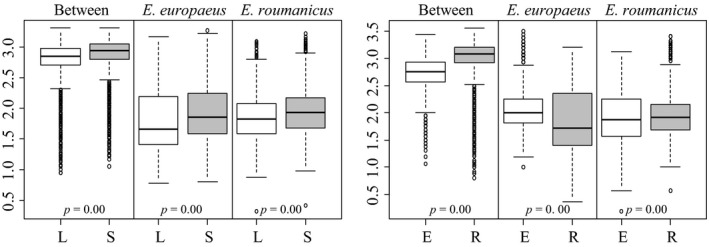
Boxplots describing pairwise distance between samples. Left panel using different allele calling approaches: sequence length (L) and sequence information (S). Right panel using different markers sets: *E. europaeus*‐specific primers (E) and *E. roumanicus*‐specific primers (R). Distance between the two species (Between) and within each species are shown. *p‐*Values correspond to *t* tests comparing differences in averages between genotyping methods and markers sets

Variability per marker was higher when sequence information was considered for allele calling (Figure 4). This difference was significant (*p* < 0.05) for all comparisons using *N*_a_ and for *H*_E_ and PIC when all samples were considered. Distance among individuals was calculated based on the average number of different alleles per marker between and within each species. Distance between species varied between 0.95 and 3.32 for length information and between 1.05 and 3.32 for sequence information. Among *E. europaeus* samples, distance ranged from 0.78 to 3.17 for length information and from 0.80 and 3.27 for sequence information. Among *E. roumanicus*, it varied between 0.32 to 3.10 for length information and between 0.41 and 3.22 for sequence information. As shown in Figure 5, distance was higher between species while no differences were found within species. Distance was also significantly higher (*p* < 0.05) when sequence information was considered (Figure 5).

Genetic diversity and marker variability were not clearly different between the two marker sets used, although the set using markers specific for *E. europaeus* were slightly more diverse (Figure 4). This was only significant when only *E. europaeus* samples were used. When the same comparison was performed using genetic distance among individuals, one of the marker sets recovered significantly higher distances than the others (Figure 5), for all test. *E. roumanicus*‐specific markers resulted in higher distances between species (Figure 5). Within species, *E. europaeus* markers contributed to a slightly higher distance among *E. europaeus *individuals. No difference between the marker sets is observed for *E. roumanicus *among the samples.

### 3.5 Genetic structure

**Figure 6:**
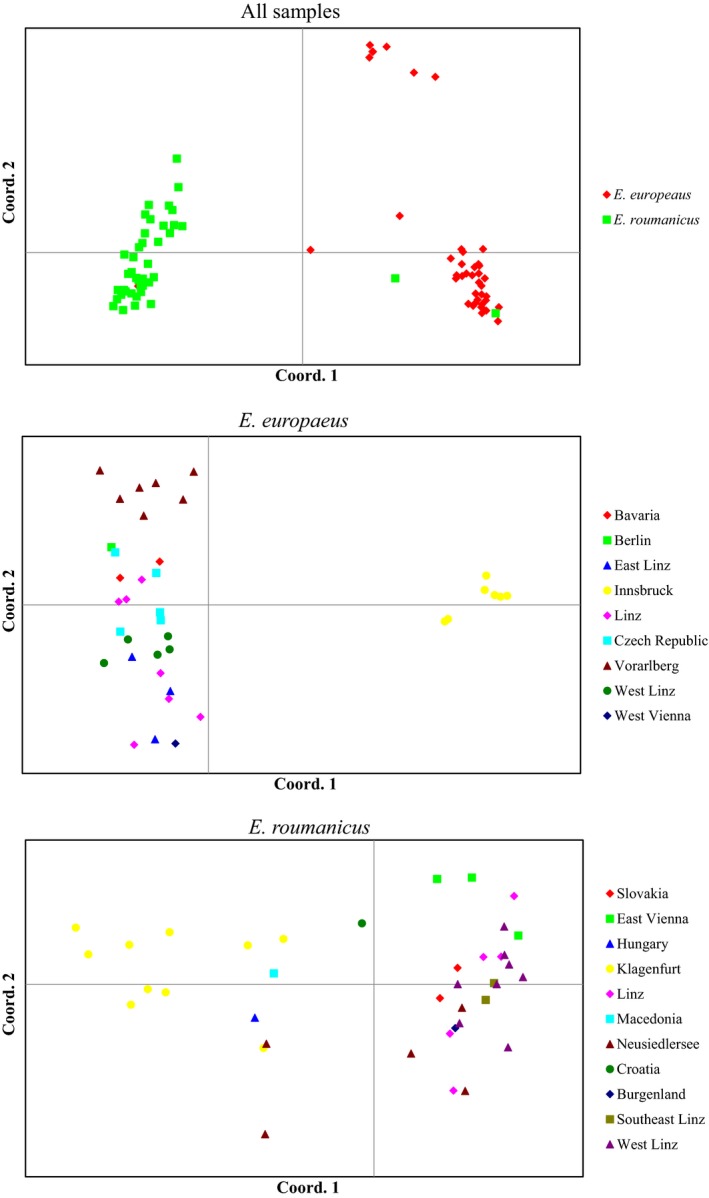
Principal coordinates analyses from matrix with genotypes called based on sequence information. Top: PCoA with complete dataset. Middle: PCoA with only *E. europaeus* samples. Bottom: PCoA with *E. roumanicus*. In the analysis for all samples, the samples are color‐coded according to species, while in the other two they were coded according to geographical region. For the analyses containing only one species samples showing ambiguous assignment in the complete dataset, PCoA were not included

When all individuals from both species were considered, the PCoA analysis resulted in two clear groups corresponding to the two species (Figure 6). There was one *E. roumanicus* individual from Linz (2016169) that appears in the *E. europaeus *group and one *E. europaeus *individual from east Linz (2014581) that groups together with the *E. roumanicus *samples. The PCoA also shows some samples that are in intermediate positions between both groups: one *E. europaeus *from Linz (2012159) and one *E. roumanicus* from the southern region of Linz (2016169). When considering only *E. europaeus* individuals, the PCoA showed three clear groups: one comprised by the samples collected by the Innsbruck shelter, another by the samples collected by the Vorarlberg shelter, and a last one containing the remaining samples. When considering only *E. roumanicus* individuals, two larger groups are found reflecting a separation between individuals from the northwestern and southeastern regions of the sampling: southeast being composed of the samples collected in the Klagenfurt shelter, Burgenland, Macedonia, Hungary, and Croatia; and the northwest containing the remaining samples. Samples from the easternmost region of Austria (Neusiedlersee) seem to be between these two groups.

**Figure 7:**
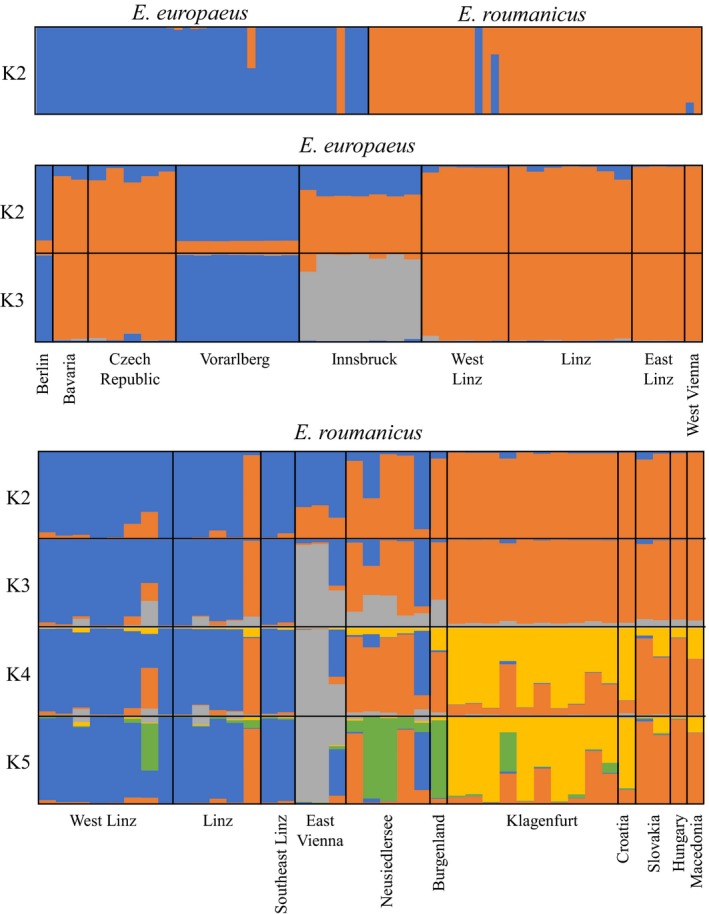
Structure analysis for all three datasets (All samples, only *E. europaeus, *only *E. roumanicus*) considering all markers and alleles called based on sequence information. Only results from *K* = 2 until the optimum are shown

STRUCTURE analyses were congruent with the PCoA results. When both species were considered, the optimal *K*‐value was two (Figure 7). For this analysis, both species were clearly separated into two clusters, with four samples showing either some degree of admixture or an opposite assignment to their morphological classification. These were the same individuals misidentified or showing signals of admixture in the PCoA analysis. The STRUCTURE analyses with only *E. europaeus *and *E. roumanicus *samples resulted in best *K*‐values of 3 and 5, respectively. Nevertheless, we also considered lower values of *K* to see if there was any congruence between the hierarchy cluster divisions and geographical distribution. For *E. europaeus, *in the *K* = 2 analysis, samples from the Vorarlberg shelter and Berlin were separated from the remaining ones. For *K* = 3, the additional cluster contains only the individuals from the shelter in Innsbruck. Considering the *E. roumanicus *dataset, for *K* = 2, one of the clusters is more prevalent in southern Austria (Klagenfurt and Burgenland) and the other countries while the other in the west (Linz region). The localities geographically between these groups (Vienna and Neusidlersee) show some degree of admixture. This pattern corresponding to a gradual transition of a cluster from southeast to another in the northeast is congruent with a scenario of isolation by distance. For the higher values of K, the following subgroups are observed: for *K* = 3, samples from Vienna are separated from the rest; for *K* = 4, the shelter from Klagenfurt has its own cluster; and for *K* = 5, it is possible to observe a new cluster comprising some samples from Neusiedlersee, the sample from Burgenland, and one individual from West Linz. For both species, although significant, there was a small correlation between geographical and genetic distance (Supporting Information Figure S1) indicating a slight signal of isolation by distance. This correlation was more pronounced for *E. europaeus* (*r* = 0.35) then *E. roumanicus* (*r* = 0.25).

Clustering results obtained with STRUCTURE, differed between the two allele calling approaches in particular for the *E. europaeus* dataset where the samples from Bavaria and Czech Republic had different assignments (Supporting Information Figure S2). Overall, allele calling based on sequence information showed a lower number of individuals with mixed assignment. When the same analysis was used to test the impact of using species‐specific primers, this resulted in a slightly clearer assignment for *E. europaeus* (Supporting Information Figure S3), while for the *E. roumanicus* dataset the marker set played no role in recovering a clearer genetic structure pattern.

## 4 DISCUSSION

In this study, we present a set of SSR markers that can be used for genotyping by sequencing of amplicons. The SSR‐GBS approach provides a significant improvement over traditional fingerprinting methods, in particular because of three factors. First, laboratory methods are highly simplified, primarily due to the ability to utilize multiplexing PCR to a higher degree than when using fragment length analysis. Second, the ability to not only capture length polymorphisms but also SNPs results in more information for allele definition when compared to electrophoresis‐based methods, resulting in higher resolution with the SSR‐GBS approach. Third, the detection of alleles as sequences decreases ambiguity when allele calls are reproduced. This facilitates the concatenation of existing with new data and the combination of different datasets. In the following sections, we review these potential improvements, beginning with the procedure details and concluding with a discussion of the prospects of compiling large datasets for genotype analyses in hedgehogs. In addition, although similar whole‐genome genotyping without available reference sequences has been previously described (Andrews et al., 2016), we highlight the potential of the current method.

### 4.1 Marker specificity

In this study, we developed primers for two closely related species, which allows for the evaluation of cross‐amplification capacity. We started with primers for *E. roumanicus,* because for this species until now no microsatellites had been developed, there exist marker sets for *E. europaeus*. When testing cross‐species amplification, we not only found null alleles, as expected (Turini et al., 2014), but also discovered loci where the repeat unit was deleted in *E. europaeus* or invariable because the allele was fixed where the repeat motif was interrupted by a SNP, and thus, variability could no longer be measured. These markers gave a positive signal after amplification, but differ in evolutionary history and variability between and within species. This confirmed the need to develop additional markers for *E. europaeus*.

Marker selection based on their variation, their source material, and their amplification success in different species result in ascertainment bias (Brandström & Ellegren, 2008). It is common practice in microsatellite genotyping to maximize variability, and this may result in an overestimation of genetic diversity or high prevalence of null alleles (Ellegren, 2000; Huang et al., 2002; Weber & Wong, 1993). This leads to an increased information content despite limited numbers of markers. When using GBS, the inclusion of additional markers does not increase the workload making this of less importance/unnecessary. Therefore, markers were not filtered based on variability. Developing marker sets based on several species minimizes ascertainment bias within each species. When biases related to the species of the marker set origin were evaluated, few differences were found in the results of genetic diversity; however, this was not the case for distance between samples and species. *Erinaceous roumanicus*‐specific markers resulted in higher differentiation between species while those specific to *E. europaeus *resulted in a higher differentiation among individuals of the same species. This difference in performance between marker sets further indicate that using only one marker set could have contributed to the presence of variability biases in our dataset.

### 4.2 Better resolution

We showed that allele call considering complete sequence information (both length and SNPs) leads to higher values for marker variability, information content, and distance between species. This improvement was most likely related to a higher number of alleles recovered with sequence information. In most cases, sequence allele definition led to an increased number of alleles and PIC, which increased anywhere from zero to 267% depending on the locus. Part of the improvement on the genetic structure may be due to the decrease in the amount of homoplasy, which is difficult to estimate with length polymorphism information alone. This was shown by the increase in singleton alleles when sequence information was used, which resulted in the division into multiple alleles of length polymorphism alleles with the same length but different nucleotide composition. However, the definition of alleles according to sequence information did not change much the overall structure assignment, likely as consequence of the high number of markers used.

The decrease in homoplasy and the large number of markers can also explain the lack of significantly higher genetic diversity using the allele calling approach for some of the comparisons made. This was the case for *H*_O_ for all tests and for *H*_E_ and PIC at an intraspecific level. Homoplasy is more likely to be found when comparing both species, so it makes sense that the genetic diversity results were significantly higher when all samples were included but not necessarily within species or populations. Given the high number of markers, most of the variation was already recovered using the length approach. Within one population, individuals are more closely related; thus, it is less likely to find homoplasy. Consequently, with sequence‐based genotyping we did not find a significantly higher genetic diversity at this level.

Studies using microsatellites on hedgehogs are currently based on two sets of markers that had been developed by Becher and Griffiths (1997) and by Henderson, Becher, Doncaster, and Maclean (2000) comprising a total of 11 loci. These markers were, in some cases, able to differentiate genetic clusters on a rather small spatial scale, which in other studies was not as pronounced (i.e., Braaker et al., 2017). For example, compared to Braaker et al. (2017), which found between 2 and 15 alleles with an average of 8, our study obtained a similar number of alleles while using only *E. europaeus *with species‐specific markers and length information (between 2 and 17 with an average of 8). These numbers increased with sequence information, ranging between 3 and 23 with an average of 11.5. We included all markers showing an amplification product, despite possibly only being informative within one species, because they can be useful for intraspecific comparisons and other similar questions. For intraspecific comparisons, we could concentrate on markers with high PIC and complement this with new loci. The high number of alleles found in some of our markers, for example, the markers W12 (50 alleles) and E23 (49 alleles), may be a consequence of gene duplication or scoring errors. Despite not finding an effect in the results, we recommend excluding them in further studies.

### 4.3 Simplification of the procedure

The laboratory methods are based on the amplicon sequencing approach suggested by Illumina and widely used for DNA bar coding (Cruaud, Rasplus, Rodriguez, & Cruaud, 2017; Shokralla et al., 2015). This approach allows a higher level of multiplexing than traditional methods, where typically up to four markers are combined in one PCR. This high number requires optimization which is only cost‐effective in studies with a large number of samples. In the current experiments, we routinely multiplexed 10 markers; however, in one experiment up to 30 markers were successfully multiplexed in one reaction. In our previous work, based on the asymmetric PCR approach (Curto et al., 2015, 2013), we used a multiplex of four markers in an electrophoresis genotyping approach, with between 4 and 5 PCRs per sample and the same number of ABI electrophoreses. In comparison, with the system presented here, we can reach this amount with one or two PCRs and comparable primer costs.

### 4.4 Better reproducibility and easier analysis

The main advantage of using SSR‐GBS is the better reproducibility of the data (de Barba et al., 2017). In traditional electrophoresis‐based determinations of SSR alleles, mobility of DNA fragments in the polyacrylamide matrix (used in most applications) is measured against an internal dye‐labeled size standard. The size of the allele is then called in comparison to the standard fragment sizes. The fragments do not always migrate through the capillary the same way, creating variation between runs, capillary sets, and laboratories (Davidson & Chiba, 2003; Fernando, Evans, Morales, & Melnick, 2001). In our experience, within one project different plates might differ by 1 or 2 bp in size estimates, which requires manual control of the range within which each allele occurs. Using tetra‐ or pentanucleotide repeats, as frequently done with vertebrates, this is generally not a problem, but with di‐ and trinucleotides this effect is more problematic due to the length ranges of possible alleles (“bins”) which are narrower for these motifs (Ginot, Bordelais, Nguyen, & Gyapay, 1996; Litt, Hauge, & Sharma, 1993). Additionally, *Taq* polymerase adds a single nucleotide to the 3′ end of the PCR product, most frequently Adenine (Brownstein, Carpten, & Smith, 1996; Magnuson et al., 1996). As a frequent artifact which is observed depending on PCR performance, this cannot be omitted and an allele may be divided into two peaks that differ by one base. The so‐called “plus A peak” artifact is a combination of this amplification artifact and variation of fragment and size standard migration in the electrical field. Ultimately, it can lead to errors of two to three base pairs, which can be further increased depending on the fluorescent dye used. The necessity for including samples of known genotype as a standard to verify allele identity is therefore common practice. As a result, the use of SNPs over SSR markers for high‐interest species data collected by multiple laboratories has been suggested (e.g., for wolfs by Kraus et al., 2015).

In SSR‐GBS, the “plus A peak” artifact is no longer relevant as the allele definition is not dependent on positions upstream of the primer binding sites, and the ambiguity that stems from electrophoresis and the addition of extra bases by the enzyme is not applicable when the fragment length is determined by the sequence composition. However, slippage artifacts may still occur with SSR‐GBS because of its’ dependency on PCR and all of the optimization procedures (Ellegren, 2004). The method is, in this respect, comparable to electrophoresis‐based methods, and therefore, ambiguities remain, especially for dinucleotide motifs.

Previous studies used primers already containing the index for sample identification and included only tetra‐ and pentanucleotide repeats to reduce PCR complexity and thus artifacts (de Barba et al., 2017). The high costs associated with this can be justified considering certain model systems such as *Ursus arctus*, a large carnivore with a high public interest, but not for small scale, non‐model organism research, for which our method would be more appropriate. To gain experience of the method's properties, we decided to include dinucleotide repeats, which are frequently used in other systems, in particular for plants (Lagercrantz, Ellegren, & Andersson, 1993; Tóth, Gáspári, & Jurka, 2000). Dinucleotides, compared to tetra‐ and pentanucleotides, have a higher probability of producing stutter bands, which are problematic for allele determination (Ginot et al., 1996; Litt et al., 1993). Nevertheless, in most cases, this limitation can be overcome during the allele call procedure.

In the dataset presented here, allele calling was not performed completely automatically. De Barba et al. (2017) presented a pipeline for automated allele calling of sequence‐based alleles (i.e., including SNPs). However, the procedure suggested did not work for dinucleotides, so a slightly different approach was chosen. First, we used the length polymorphisms to determine the SSR allele, that is the most likely allele definition according to length, and thus the repeat unit number. In a second step, we investigated whether the SSR allele contained additional single nucleotide polymorphisms or not. Similar to traditional electrophoresis‐based analysis, this approach is very accurate for tetra‐ and pentanucleotide repeats, but has a higher error rate with dinucleotides. Here, the difficulty in determining alleles when stutter bands of one allele overlay another still exists because the determination of the SSR allele is performed according to length frequency distribution and does not differ in this respect from traditional analyses. When both alleles differ on base composition, this overlay applies also to SNPs, which means that an SSR allele overlaid by a stutter band can show a nucleotide polymorphism as an artifact. Here, the state of the other allele must be taken into consideration. The approach of de Barba et al. (2017) is also unable to overcome this limitation since it divides alleles based on SNPs in the flaking regions first. The program HipSTR (Willems et al., 2017) can deal with the stutter effect by using a parametric approach. It defines candidate alleles based on a stutter model and uses them as reference to align the reads redefining new candidate alleles. This process is repeated until the most likely alignment is obtained. Since this approach is based on alignment quality, it is likely to be negatively affected by erroneous phasing between SNP variations in the flanking regions and the repetition motif. As mentioned above, this can be caused by the overlay of stutter bands and the formation of chimeric sequences in the PCR. These artifacts result in sequences containing the repetition motif of one allele and the SNP variant of the other. HipSTR does not have a filtering step where these error sources are considered, and thus, all sequences stemming from PCR artifacts are included during allele call. This can potentially contribute to a lower likelihood of alignments of the correct alleles. In our method, because we filter out reads first based on length, with a manual control step, a lot sources of error are already excluded, decreasing the ambiguity of the final allele calling. There are alternative approaches based on the assembly of the amplicon reads. Šarhanová, Pfanzelt, Brandt, Himmelbach, and Blattner (2018) applied an alternative approach based on read de novo assembly. Nevertheless, a manual control step was added to account for the assembly of two alleles filtering noise. Thus, at this moment, a manual curation step is still necessary in the genotyping of di‐ and trinucleotides repeats.

The high reproducibility that can be achieved in determining sequence alleles also allows for the easy creation of large data collections over multiple laboratories and projects. There are several examples where SSR variation is used for wildlife monitoring; however, the technical difficulties restrict this to species for which there is considerable conservation concern (Godinho et al., 2011), conflict species (De Barba et al., 2010), or species with large commercial interest (Schenekar & Weiss, 2017; Tibihika, Waidbacher et al., 2018). With similar approaches to the SSR‐GBS system, this can be adapted for non‐model species and specific scientific questions. Our interest in hedgehogs resulted from a citizen science project, where occurrence data had been collected in private gardens together with that from primary school students and the general public. The prospect of including methods that allow for investigation of a variety of samples, using hair, feces, or mouth swabs is very interesting and could be achieved by the SSR‐GBS system presented here. In our case, although mouth swabs showed higher missing data than tissue samples this did not affect the final results. This was a consequence of lower number of reads for these samples. The potential of SSR‐GBS can be compared to phylogenetic data collections, where sequences can very easily be incorporated into existing alignments and large meta‐analyses are frequent (Adams, 2008). It therefore constitutes a tool that can be implemented in long‐term screening projects.

### 4.5 Phylogeographic implications

Two of the included individuals were detected as potential hybrids. Using a dense sampling from the contact zone in the Check Republic, Bolfíková and Hulva (2012) did not find any evidence of hybridization among the two hedgehog species. However, hybridization among these species would be congruent with the high incidence of hybrid zones in central Europe (Hewitt, 2001). The current rarity of hybridization events can be a remnant of a hybrid zone dynamics. It is likely that every time these species contacted after a glacial period a hybrid zone was established. With time, these species may have become more reproductively isolated to a point that the hybrid zone either only exists in some areas or it is very narrow. This hypothesis can only be tested by characterizing hybridization occurrence and frequency across the contact zone.

Overall there was a weak correlation between genetic structure and geographical distance, which may be a consequence of barrier to gene flow, promoted by natural and anthropogenic factors. For example, there was a separation among *E. roumanicus* individuals from the south and north of the alps indicating that these mountains may work as a natural barrier. Additionally, human structures such as roads may have contributed to some structure found at the local level (Braaker et al., 2017). This has been reported to be the case for *E. europaeus* populations in England (Becher and Griffiths 1998). The potential role of natural anthropogenic structures on hedgehog populations from central Europe needs to be better investigated with a denser sampling in order to account for small scale genetic structure as well.

Shelters’ practices may also influence the distribution of genetic variability. This happens when the source of the individuals are unknown and they are consequently not released in areas of their origin. This may contribute to outbreeding depression and promote hybridization (Edmands, 2007). In the current study, the individuals from the shelters are genetically homogeneous, so as long as the shelter does not release individuals outside the area of activity the gene pool of natural populations should not be affected. Given the low amount of shelters and limited sampling, it is still impossible to make any conclusion in this matter and we are currently in the process of including a larger sampling from multiple shelters spread throughout Central Europe.

### 4.6 Importance of the museum collections

The improvement of replicability associated with the SSR‐GBS approach may allow several long‐term studies using newly collected and museum samples. For our study, we were able to utilize a large collection of hedgehog specimens preserved in ethanol at the Biologiezentrum in Linz. This emphasizes the usefulness of the storage of multiple samples, especially from species that attract public attention, by public collections. In the Biologiezentrum Linz, this was achieved by combining several private collections with staff efforts, from which studies like this one benefit. This also demonstrates how desirable it is to store multiple samples per species even if space problems and considerations of general funds might suggest otherwise. This is especially true when, like in the present dataset, potential hybrids are found and the determination of morphological characters may be critical to complement the molecular data.

## CONFLICT OF INTEREST

None declared.

## AUTHOR CONTRIBUTION

The Experiment was planned and designed by HM and MC. MC and SW wrote the bioinformatics pipeline with contributions of KS. Genotypes were analyzed by MC, HM, LS, and AS. LS and AS part of the dataset as in the context of their master theses. LB and JP provided and organized the samples. HM and MC led the writing of the manuscript with contributions of all authors.

## Data Availability

Raw reads from the low‐coverage whole‐genome sequencing libraries used for marker development can be found in the Sequence Read Archive (SRA) under the reference PRJNA495814. The SSR allele sequences were submitted to GenBank and can be found with the reference numbers MH683170‐MH683548.
