# Supplementary material for: Application of a SSR‐GBS marker system on investigation of European Hedgehog species and their hybrid zone dynamics
Source: Ecol Evol. 2019 Feb 14;9(5):2814–32. doi: 10.1002/ece3.4960 (PMC6405497; doi:10.1002/ece3.4960)
Supplement: Supplementary file 1 [file ECE3-9-2814-s001.docx]

**Supplementary Material**

Table S1. Samples used with information of location of origin, species identification, starting material for DNA isolation, institution providing the sample, and coordinates. For some samples coordinates were not available (NA).

| **Sample Name** | **Region** | **Species** | **Material** | **Material origin** | **Coordinate** |
| --- | --- | --- | --- | --- | --- |
| 36516 | Berlin | *E. europaeus* | tissue | Leibniz Institute for Zoo and Wildlife Research | NA |
| 2014429 | Bavaria | *E. europaeus* | tissue | Biologiezentrum Linz | 48.268194, 13.034333 |
| 2014430 | Bavaria | *E. europaeus* | tissue | Biologiezentrum Linz | 48.268194, 13.034333 |
| 200689 | Czech Republic | *E. europaeus* | tissue | Biologiezentrum Linz | 48.972278, 14.473722 |
| 2006603 | Czech Republic | *E. europaeus* | tissue | Biologiezentrum Linz | 48.735556, 14.491667 |
| 2008188 | Czech Republic | *E. europaeus* | tissue | Biologiezentrum Linz | 48.814528, 14.284139 |
| 20111185 | Czech Republic | *E. europaeus* | tissue | Biologiezentrum Linz | 48.846472, 14.455722 |
| 20111186 | Czech Republic | *E. europaeus* | tissue | Biologiezentrum Linz | 48.786028, 14.456222 |
| 2004247 | West Linz | *E. europaeus* | tissue | Biologiezentrum Linz | 48.343861, 14.720444 |
| 2005615 | West Linz | *E. europaeus* | tissue | Biologiezentrum Linz | 48.243000, 14.849889 |
| 2006606 | West Linz | *E. europaeus* | tissue | Biologiezentrum Linz | 48.358806, 14.511944 |
| 2007102 | West Linz | *E. europaeus* | tissue | Biologiezentrum Linz | NA |
| 2012159 | West Linz | *E. europaeus* | tissue | Biologiezentrum Linz | 48.141556, 13.735889 |
| 2016172 | West Linz | *E. europaeus* | tissue | Biologiezentrum Linz | 48.336861, 14.435667 |
| 2002243 | Linz | *E. europaeus* | tissue | Biologiezentrum Linz | 48.337944, 14.311972 |
| 2014439 | Linz | *E. europaeus* | tissue | Biologiezentrum Linz | 48.336806, 14.313083 |
| 2014456 | Linz | *E. europaeus* | tissue | Biologiezentrum Linz | 48.331750, 14.312806 |
| 2014839 | Linz | *E. europaeus* | tissue | Biologiezentrum Linz | 48.334111, 14.326778 |
| 2015786 | Linz | *E. europaeus* | tissue | Biologiezentrum Linz | 48.334111, 14.326778 |
| 200695 | Linz | *E. europaeus* | tissue | Biologiezentrum Linz | 48.334111, 14.326778 |
| 200792 | Linz | *E. europaeus* | tissue | Biologiezentrum Linz | 48.332389, 14.312806 |
| 2008219 | East Linz | *E. europaeus* | tissue | Biologiezentrum Linz | 48.345444, 14.511917 |
| 2014438 | East Linz | *E. europaeus* | tissue | Biologiezentrum Linz | 48.254750, 14.424056 |
| 2014581 | East Linz | *E. europaeus* | tissue | Biologiezentrum Linz | 48.351444, 14.092444 |
| 2014683 | East Linz | *E. europaeus* | tissue | Biologiezentrum Linz | 48.322167, 14.194278 |
| 2016171 | East Linz | *E. europaeus* | tissue | Biologiezentrum Linz | 48.361806, 14.490889 |
| 2014445 | West Vienna | *E. europaeus* | tissue | Biologiezentrum Linz | 48.126806, 14.595306 |
| IBK1 | Innsbruck | *E. europaeus* | saliva | shelter Innsbruck | NA |
| IBK2 | Innsbruck | *E. europaeus* | saliva | shelter Innsbruck | NA |
| IBK3 | Innsbruck | *E. europaeus* | saliva | shelter Innsbruck | NA |
| IBK4 | Innsbruck | *E. europaeus* | saliva | shelter Innsbruck | NA |
| IBK5 | Innsbruck | *E. europaeus* | saliva | shelter Innsbruck | NA |
| IBK6 | Innsbruck | *E. europaeus* | saliva | shelter Innsbruck | NA |
| IBK7 | Innsbruck | *E. europaeus* | saliva | shelter Innsbruck | NA |
| VA25 | Voralberg | *E. europaeus* | saliva | Sheter Bludenz | NA |
| VA26 | Voralberg | *E. europaeus* | saliva | Sheter Bludenz | NA |
| VA27 | Voralberg | *E. europaeus* | saliva | Sheter Bludenz | NA |
| VA28 | Voralberg | *E. europaeus* | saliva | Sheter Bludenz | NA |
| VA29 | Voralberg | *E. europaeus* | saliva | Sheter Bludenz | NA |
| VA30 | Voralberg | *E. europaeus* | saliva | Sheter Bludenz | NA |
| VA31 | Voralberg | *E. europaeus* | saliva | Sheter Bludenz | NA |
| 2008174 | Slovakia | *E. roumanicus* | tissue | Biologiezentrum Linz | 48.669667, 17.782222 |
| 2008176 | Slovakia | *E. roumanicus* | tissue | Biologiezentrum Linz | 48.619194, 20.634222 |
| 2014420 | Croacia | *E. roumanicus* | tissue | Biologiezentrum Linz | 47.770000, 16.801306 |
| 2014417 | Hungary | *E. roumanicus* | tissue | Biologiezentrum Linz | 47.629778, 16.636306 |
| 201463 | Macedonia | *E. roumanicus* | tissue | Biologiezentrum Linz | 44.061111, 18.589667 |
| 200674 | West Linz | *E. roumanicus* | tissue | Biologiezentrum Linz | 48.227417, 14.600472 |
| 2006187 | West Linz | *E. roumanicus* | tissue | Biologiezentrum Linz | 48.203833, 14.731222 |
| 2008185 | West Linz | *E. roumanicus* | tissue | Biologiezentrum Linz | 48.338028, 14.298750 |
| 2009167 | West Linz | *E. roumanicus* | tissue | Biologiezentrum Linz | 48.343083, 14.702861 |
| 2014565 | West Linz | *E. roumanicus* | tissue | Biologiezentrum Linz | 48.250528, 14.580056 |
| 2014582 | West Linz | *E. roumanicus* | tissue | Biologiezentrum Linz | 48.215444, 14.449528 |
| 2014837 | West Linz | *E. roumanicus* | tissue | Biologiezentrum Linz | 48.228361, 14.528750 |
| 2016168 | West Linz | *E. roumanicus* | tissue | Biologiezentrum Linz | 48.189111, 14.696389 |
| 200669 | Linz | *E. roumanicus* | tissue | Biologiezentrum Linz | 48.298472, 14.303611 |
| 200675 | Linz | *E. roumanicus* | tissue | Biologiezentrum Linz | 48.279750, 14.389139 |
| 2006613 | Linz | *E. roumanicus* | tissue | Biologiezentrum Linz | 48.282972, 14.287611 |
| 2008184 | Linz | *E. roumanicus* | tissue | Biologiezentrum Linz | 47.874333, 16.945667 |
| 2014425 | Linz | *E. roumanicus* | tissue | Biologiezentrum Linz | 48.327444, 14.327389 |
| 2016169 | Linz | *E. roumanicus* | tissue | Biologiezentrum Linz | 48.313000, 14.276139 |
| 2012154 | East Vienna | *E. roumanicus* | tissue | Biologiezentrum Linz | 48.279056, 16.635917 |
| 2012155 | East Vienna | *E. roumanicus* | tissue | Biologiezentrum Linz | 48.279056, 16.635917 |
| 04NHMro | East Vienna | *E. roumanicus* | tissue | Natural History Museum | NA |
| 2008186 | Southeast Linz | *E. roumanicus* | tissue | Biologiezentrum Linz | 48.029833, 14.189528 |
| 2014427 | Southeast Linz | *E. roumanicus* | tissue | Biologiezentrum Linz | 48.042667, 13.989028 |
| 2014838 | Southeast Linz | *E. roumanicus* | tissue | Biologiezentrum Linz | 48.202389, 14.119889 |
| 2008182 | Neusidlesee | *E. roumanicus* | tissue | Biologiezentrum Linz | 46.435667, 15.904111 |
| 2013168 | Neusidlesee | *E. roumanicus* | tissue | Biologiezentrum Linz | 47.723167, 16.867111 |
| 2014421 | Neusidlesee | *E. roumanicus* | tissue | Biologiezentrum Linz | 47.770000, 16.801306 |
| 2014422 | Neusidlesee | *E. roumanicus* | tissue | Biologiezentrum Linz | 47.742889, 16.832861 |
| 2014423 | Neusidlesee | *E. roumanicus* | tissue | Biologiezentrum Linz | 48.206361, 14.380111 |
| 2015109 | Burgenland | *E. roumancius* | tissue | Biologiezentrum Linz | 47.060361, 16.315056 |
| KLF66 | Klagenfurt | *E. roumanicus* | saliva | Shelter Klagenfurt | NA |
| KLF67 | Klagenfurt | *E. roumanicus* | saliva | Shelter Klagenfurt | NA |
| KLF70 | Klagenfurt | *E. roumanicus* | saliva | Shelter Klagenfurt | NA |
| KLF72 | Klagenfurt | *E. roumanicus* | saliva | Shelter Klagenfurt | NA |
| KLF73 | Klagenfurt | *E. roumanicus* | saliva | Shelter Klagenfurt | NA |
| KLF76 | Klagenfurt | *E. roumanicus* | saliva | Shelter Klagenfurt | NA |
| KLF80 | Klagenfurt | *E. roumanicus* | saliva | Shelter Klagenfurt | NA |
| KLF82 | Klagenfurt | *E. roumanicus* | saliva | Shelter Klagenfurt | NA |
| KLF83 | Klagenfurt | *E. roumanicus* | saliva | Shelter Klagenfurt | NA |
| KLF84 | Klagenfurt | *E. roumanicus* | saliva | Shelter Klagenfurt | NA |

**Table S2.** Complete list of all primers designed with the following information: from which species they were designed (Species), in which primer mix they were included in the multiplex PCR (Mix), repetition motif (Motif), number of times it was repeated in the original sequence (Nr. Repeats), primer sequence (Forward and Reverse), and allele length variation (Amplicon length variation).

| **Primer Name** | **Species** | **Mix** | **Motif** | **Nr. Repeats** | **Forward** | **Reverse** | **Amplicon length variation** |
| --- | --- | --- | --- | --- | --- | --- | --- |
| E1 | *E. roumanicus* | R1 | AC | 16 | TCATGCTAGGCACTGCTATT | AAGTGCAATCAGACCAGTGA | 454 - 486 |
| E11 | *E. roumanicus* | R1 | AAAG | 7 | ACGTTCCTCTCTGGGGAATA | TTCAAGACCCTGTTCTCCAC | 428 - 460 |
| E18 | *E. roumanicus* | R1 | ATTTT | 9 | TAGCCTGGGGGAAAATCAAG | GCAATTTCCAGTAGAGGGGA | 438 - 475 |
| E26 | *E. roumanicus* | R1 | CAA | 10 | TTAAGGAACTCAGGGTTGGG | GTGTCAATGGAAGCAAAGCT | 487 - 502 |
| E29 | *E. roumanicus* | R1 | TCAA | 7 | CTTGTGCACTGTGATGTGAG | ACGAAGTTTCCAGGAAGCTC | 486 - 494 |
| E31 | *E. roumanicus* | R1 | AACA | 7 | GGAAGCGCCTTCATTATAGC | CTCCTGTCACTAGCCAGAAG | 476 - 484 |
| E36 | *E. roumanicus* | R1 | GAAAG | 9 | ACAGTGAAGACAGGGAAGC | CTTAAAATGGCTAAGGTGGT | 452 - 517 |
| E4 | *E. roumanicus* | R1 | CT | 13 | TCAAGGAGTGTGTTGACCAG | ATCCCTTTGCTCAGCCAAT | 452 - 462 |
| E9 | *E. roumanicus* | R1 | ATT | 13 | GTTGACACTCTTTGCTGCTT | CAAGTCCTCACTAAGCCTGT | 425 - 444 |
| E10 | *E. roumanicus* | R2 | AAAG | 11 | AAGCACAACAACAATGGCAA | ACGTACTGAGCCTTTCAAGA | 437 - 545 |
| E16 | *E. roumanicus* | R2 | AAGAA | 12 | CACTAGGCAGAAAACACACG | ACCACAGATGCTGTAGACAG | 425 - 480 |
| E2 | *E. roumanicus* | R2 | AC | 16 | TGGGTAGCAGCTAAAGGAAG | GACAAAATCCTCCCTGGCTA | 448 - 482 |
| E27 | *E. roumanicus* | R2 | TTG | 9 | AGATGCTCAAGGGAAACTGA | TCACAGCATACTTAGGAGCC | 452 - 477 |
| E30 | *E. roumanicus* | R2 | TCAC | 8 | AGCGTTAAATACATCCGGCT | AACCCATTGACTCTCTGACA | 430 - 458 |
| E34 | *E. roumanicus* | R2 | TCTA | 10 | AGACCACAGTGTCCAAGTTT | GATTTCCCCTGTGTAGGTGA | 428 - 466 |
| E35 | *E. roumanicus* | R2 | AAAAT | 6 | TGGGTTGTAGATAACACTCA | ACTGCAGGTGGAGATATGTG | 464 - 482 |
| E37 | *E. roumanicus* | R2 | CTTTT | 7 | CTGCAGTTTGCTCTGGATTC | AAGAAAGAAGCCCTTGTCCA | 426 - 456 |
| E5 | *E. roumanicus* | R2 | GA | 17 | TTTCTTGCTCAGAACCCTGA | CAGGGGGAATGCTTTTCAAG | 446 - 480 |
| E7 | *E. roumanicus* | R2 | AAT | 10 | ACCATAGCTTTGTAATCTCCT | AGGATGATGGCCCTTTGAAA | 445 - 463 |
| E13 | *E. roumanicus* | R3 | AAGG | 11 | AGGTAGAAGGCAGACAATGG | TTGAAACACTGACGTAGGCT | 428 - 476 |
| E19 | *E. roumanicus* | R3 | GAAAT | 12 | CCTTGCTTGTTTCCTAAGCC | ATCACTGGGACTCCCTCAAT | 472 - 527 |
| E20 | *E. roumanicus* | R3 | TATTC | 11 | TGGATGGATGGAAAACTGAGA | GGGTGCTGATTCATCTACCT | 477 - 522 |
| E22 | *E. roumanicus* | R3 | AG | 15 | ACGGAAGGAAATACTGCCAA | CCTTCCCCTTTGTGAGAACT | 470 - 502 |
| E23 | *E. roumanicus* | R3 | CCT | 8 | GGCTATAGGCAGATTGGTGT | GAAGGTCCCAGGAACCATAG | 441 - 504 |
| E24 | *E. roumanicus* | R3 | GCA | 7 | TTCTGAGGTCTCATCTGTGC | CTGTCTGTGGTCCAGGAAAG | 452 - 452 |
| E28 | *E. roumanicus* | R3 | TTG | 8 | CCTAGTGGTAGCTTCTCACA | TTGGCTCCAGTCAAGTTCTC | 440 - 452 |
| E3 | *E. roumanicus* | R3 | CA | 17 | GGCAGACTGTCTAGTTCACA | GGTCTAGGACTGCACCATTT | 476 - 512 |
| E8 | *E. roumanicus* | R3 | ATT | 8 | CCTCCAGGAGAGATTTTGCT | CAAATGAGTGGAAGCCATGC | 489 - 498 |
| W1 | *E. europeaus* | E1 | AAAAT | 7 | GGGTAAAACAGGTCTGATGT | AAACTTGTCAGGAAGCAGTT | 382 - 407 |
| W10 | *E. europeaus* | E1 | AAAAC | 7 | ATAGCTGGATAGTGGTCTGG | ACATCTTTTCTTCCTCACAGT | 398 - 433 |
| W11 | *E. europeaus* | E1 | CTTC | 10 | AGTCACCATTCTCCACTTTC | ACCCTGAGTGAAGAAGGATA | 413 - 435 |
| W12 | *E. europeaus* | E1 | GAAA | 8 | AACTCAAATTACAAGGGGCC | TCCAATAACTAGGGGTTTAAGT | 386 - 474 |
| W13 | *E. europeaus* | E1 | TTTA | 7 | TTTCACTCTGGGTTACTGTG | AAGTGGTGCAACTCTAAGAC | 386 - 395 |
| W14 | *E. europeaus* | E1 | ATAG | 10 | AAAAGGACCTAAATGGGAGG | ACAGGGAACAAAGATGCTTA | 376 - 408 |
| W15 | *E. europeaus* | E1 | ATAA | 8 | ATACTCCCAGCCTGTTTCTA | ACCTCCCAAGAACTCTATCA | 367 - 390 |
| W16 | *E. europeaus* | E1 | TTAA | 7 | GTGTAAAGCAGTATGTTGCC | AATACAGTGTACAAGGACGC | 407 - 419 |
| W18 | *E. europeaus* | E1 | AATA | 8 | ACTCAAAAGTTTTCCACCCT | TTTTAGGCTCTGCTCTTCTG | 403 - 411 |
| W19 | *E. europeaus* | E1 | TTCT | 13 | AGAGATCAGACTAACGTTTTT | GGGGAGAATTTGGTACTGTA | 402 - 443 |
| W21 | *E. europeaus* | E1 | TTTA | 7 | ACTTCACTATCACCCTTCAA | ACTTGATTTGTTTATGGGGTG | 395 - 403 |
| W23 | *E. europeaus* | E1 | TGGA | 13 | TCTTCCCTTAAGCTACTGGA | TCTCAATTGTTTAGACATTGAGT | 386 - 414 |
| W29 | *E. europeaus* | E1 | CT | 15 | CATTACCGTGCACACAGA | GTTTGATCCCCACCACTTAA | 406 - 422 |
| W30 | *E. europeaus* | E1 | CT | 17 | TCTCATTGGATAGTGCACTG | TGCCTAATAGCAAATACACA | 405 - 441 |
| W31 | *E. europeaus* | E1 | GA | 20 | CACTTTCAATGCAGAACGTG | CAAACTGGACTAGGACAGAG | 397 - 423 |
| W32 | *E. europeaus* | E1 | GT | 13 | CAGTCAATGCATTCCCAATC | TGTGTGGTACAGGGAATAGA | 415 - 451 |
| W33 | *E. europeaus* | E1 | CA | 11 | AGAAAAGACCTCAGGAGACT | CCTGGAGAGTGGAAAAGTTA | 424 - 456 |
| W6 | *E. europeaus* | E1 | TTATT | 7 | AGGAGTTCTCAGTGATGAGA | AATACAGGCTCTGGGATAGT | 378 - 404 |
| W7 | *E. europeaus* | E1 | TCTTT | 9 | TTAGCTTGGTTTTCACAGGT | GAGTGGCAGTCTTCAAGTAG | 384 - 419 |
| W8 | *E. europeaus* | E1 | TTCCT | 10 | ATAGGAGGACTGGCGATC | AATGGAGGGAGTAGATGGG | 364 - 424 |
| W9 | *E. europeaus* | E1 | TTTCT | 10 | TTCAATCTCAAGTACCACATT | GATGCACCTGGTTGAGAG | 384 - 414 |
| E32 | *E. roumanicus* | R3 | ATCT | 7 | TGACAGTGTGTGGTTGACTT | TTCACCATCGCAGAGAACAT | Failed in Multiplex |
| E25 | *E. roumanicus* | R1 | TAC | 9 | TGTTATCATGCCTGAGGACC | CTGGTTGGGAAGAGAAACCT | Failed in Multiplex |
| E6 | *E. roumanicus* | R1 | AAT | 16 | CTCTTGGTGTGCATGACAAG | CTGTGACCCGTGTAGTTGG | Failed in Multiplex |
| W20 | *E. europeaus* | E1 | TAGA | 8 | TGCACATTACAATGTTCAAGG | TACATCAGGGAGAGTACAGG | Failed in Multiplex |
| W24 | *E. europeaus* | E1 | ATA | 13 | GCAATAATAACAAGAAGGGCA | AAGAAGTGACTGGTTTGGAG | Failed in Multiplex |
| W25 | *E. europeaus* | E1 | TTA | 14 | CTTTATGGGGTGCAGAAGAT | CACGATGAGCAAAGCTATTC | Failed in Multiplex |
| W26 | *E. europeaus* | E1 | TAT | 15 | TTTCCAGAAGATGTGGTCAG | TACAAATCTCAGCACCACTC | Failed in Multiplex |
| W27 | *E. europeaus* | E1 | ATA | 9 | AGCCAAAGAATAGAAGCAAGA | GCATTCTGTGGTCATGAGTA | Failed in Multiplex |
| W3 | *E. europeaus* | E1 | AAAGA | 6 | GAAGAAGTTTCCTCCTCTGG | GGTGGACTGAACCATTTCTT | Failed in Multiplex |
| W5 | *E. europeaus* | E1 | AAAAT | 8 | CACCAGGTTAAGCGTACATA | AAAAGTGCTACTAGGGAAGC | Failed in Multiplex |
| E12 | *E. roumanicus* | Failed in single PCR | AAAG | 7 | AACAGAACAGCCCTGATGTT | TTGTCTTGCTTCTGGTGAGT | Failed in single PCR |
| E14 | *E. roumanicus* | Failed in single PCR | AAGG | 13 | ACATGACTGTGGGTTGAGTG | CTCCAGCTCCACTGCTTTAG | Failed in single PCR |
| E15 | *E. roumanicus* | Failed in single PCR | AAATA | 7 | CTGGATCAGTGAAGCTTCCA | CAAACTGGGTTAAGTGCACA | Failed in single PCR |
| E17 | *E. roumanicus* | Failed in single PCR | ATTCC | 18 | ACAACCCTTCAGCTTCATCA | TAGTAGGGTGAGTCTCTGGG | Failed in single PCR |
| E21 | *E. roumanicus* | Failed in single PCR | GA | 14 | TACCCATTATGCTACCCACC | TTCTGGTACATGTGCTACCG | Failed in single PCR |
| E33 | *E. roumanicus* | Failed in single PCR | ATCT | 13 | CTCCATCACATGTGCCAAAG | CCACTGGCATACTACTGTGT | Failed in single PCR |
| W17 | *E. europeaus* | Failed in single PCR | GAAA | 9 | TGTGATGAGGTGTTTGTTCT | AGATTTGTTGCAGGTGTCTC | Failed in single PCR |
| W2 | *E. europeaus* | Failed in single PCR | AAATA | 8 | CATGAATCCACTGCTCCTAG | CTGTAGAGGTGTTGTTTTGC | Failed in single PCR |
| W22 | *E. europeaus* | Failed in single PCR | AGGA | 14 | CTCATTGCAGGAAACTTCAC | GTTGTATTGTCTTATTTGGAGGT | Failed in single PCR |
| W28 | *E. europeaus* | Failed in single PCR | AC | 16 | TTCTTGTTAGACCCTGAAGC | GTGACACTGGGACTCAAAC | Failed in single PCR |
| W34 | *E. europeaus* | Failed in single PCR | AG | 15 | AGGGAACTGCACTATGTCTA | GCACACCTGGTTAAACACAT | Failed in single PCR |
| W4 | *E. europeaus* | Failed in single PCR | TTTTG | 7 | ACTGAAGGAAGCTTCTGTG | GTAGTCTTTGAGCTTTGTGC | Failed in single PCR |

**Table S3**. Descriptions and procedures used in the in-house-scripts. We used the scripts one after the other and information concerning how this was done can be found in the material and methods. The scripts 1 to 3 can be run sequentially using the wrapper *microsatPip*.

**microsatPip**

**File name:**  microsatPip.sh

**Requirements**: Unix, scripts 1 and 3 in the same directory of microsatPip.sh

**Description:** This is wrapper script that runs the programs FASTQC, PEAR, plus the scripts 1 to 3 one after the other. The results from each step are saved in a directory containing the seven folder. Initially the it runs the program FASTQC where the output is saved in the folder “FastqcOut”. The it sorts the read according to name saving them in the directory “Sorted”. Sorted reads are then merged with PEAR and saved in the folder “MergedOut”. Files are renamed based on a user inputted sample sheet. Samples names should not contain any spaces or underscores. The renamed files are saved in the folder “SeparatIn” and used for demultiplexing. Demultiplexed fastq files are saved in the folder “SeparatOut”. Sequence lengths counts are determined and saved in one text file per sample per markers in the directory “MarkerStatistics”. The csv codominant matrix and sequence length plots produced by script 3 are saved on the “Markerplots” directory.

**How to run: ./**microsatPip.sh [1] [2] [3] [4] [5]

extract_reads_correct_primer_merged.py [1] [2] [3] [4] [5]

[1] Directory containing input fastq files.

[2] Quality threshold from which sequences should be filtered in quality control step.

[3] File containing primer information: this should be a tab separated text file containing one locus per line with the following information:

maker name[TAB]sequence primer forward[TAB] sequence reverse primer

Markers should be named in the following way: MarkerName_RepetitionMotif (ex: HH1_AT)

[4] File containing sample names information: this should be a coma separated file containing one sample per line with the following format: Name in input fastq before the first underscore , new name.

For example, for the paired reads files: P5-1-P7-1_L001_R1_001.fastq, P5-1-P7-1_L001_R2_001.fastq to be replaced with the name Sample1, this should have the following format:

P5-1-P7-1,Sample1

| **Script number 1**  **File name:**  primer_demultiplex.py  **Requirements**: python 2 or 3; Biopython  **Description:** This script demultiplexes merged fastq files according to primer content. The outputs are one fastq file per sample and per locus. This script allows for a user specified maximum number of mismatches between the primer and the reads. Only reads with a mismatch to both primers below to the defined are kept. In this case they are saved in a separate file. Moreover, sequences below a certain length can be excluded.  **How to run:** python extract_reads_correct_primer_merged.py [1] [2] [3] [4] [5]  [1] Directory containing input fastq files. Files should be named without the underscored character (ex: Sample1.fastq)  [2] File containing primer information: this should be a tab separated text file containing one locus per line with the following information:  maker name[TAB]sequence primer forward[TAB] sequence reverse primer  Markers should be named in the following way: MarkerName_RepetitionMotif (ex: HH1_AT)  [3] Maximum number of mismatches  [4] Minimum sequence length  [5] Directory to save output files. Files names are save in the following format: RepetitionMotif_SampleName_MarkerName.fasq (ex: Sample1_HH1_AT.fastq) |
| --- |

**Script number 2**

**File name:** CountLengths.sh

**Requirements**: Unix system

**Description:** Per fastq file it counts the number of occurrences of each sequence length present. It outputs this information in a space separated text file being the first column the length and the second the number of occurrences. Example:

417 14

422 18

418 276

423 282

**How to run:** sh CountLengths.sh [1] [2]

[1] Directory containing input demultiplexed fastq files. Files should be as described in the output from script 1: RepetitionMotif_SampleName_MarkerName.fasq (ex: Sample1_HH1_AT.fastq)

[2] Directory to save output files. Output file is saved in the following format: MarkerName_RepetitionMotif_SampleName_.statistics (ex: HH1_AT_Sample1_.statistics)

| **Script number 3**  **File name:** Rscript_Markerlength_develop_Color.R  **Requirements**: R version 3.4.1 or newer, packages reshape and reshape2 (Wickham 2007) will be installed automatically by the R script.  **Description:** This script uses sequence length counts to call alleles and define genotypes. A homozygote genotype is considered if the relative read count of the most abundant allele is larger than the user defined alpha value (e.g. 0.7). A heterozygote genotype is considered when the sum of the relative abundance of two most abundant read lengths exceeds the user defined alpha value  (i) and if the difference in length of the potential alleles is larger than one time the repeat motif length.  (ii) and if the two most abundant potential alleles only differ in one repeat size length, the less abundant allele is considered true if it is longer than the most abundant allele.  (iii) and if the second largest potential allele is just one time smaller than the repeat motif length, it is only considered if its relative abundance equals 0.75 of the most abundant allele.  (iv) in addition, we also considered point mutations which lead to non-integer multiples of the repeat length, if their relative abundance was at least 0.6 of the most abundant allele.  If these criteria are not met the sample is considered for manual control. Genotypes are saved in a coma separate file and length histograms are plotted in a pdf file highlighting the called alleles with different colors: blue for the homozygous allele, green for the alleles in heterozygous samples and black for the other sequence lengths.  **How to run:** Rscript --vanilla Rscript_Markerlength_develop_Color.R [1] [2] [3] [4] [5] [6] [7] [8]  [1] Directory containing length counts. This is the output from script 2.  [2] Minimum count of the total reads for a genotype to be considered.  [3] Path to directory where pdf file with length counts histograms will be saved.  [4] Alpha threshold for defining homo- and heterozygotes genotypes.  [5] Path to Csv matrix file containing genotypes of markers of the respective samples  [6] Path to Csv file containing three columns with sample and marker name and respective allele  [7] Minimum allele size length for plotting the results  [8] Maximum allele size length for plotting the results  **References**  Wickham, H. (2007). Reshaping Data with the reshape Package. Journal of Statistical Software, 21(12), 1-20. URL <http://www.jstatsoft.org/v21/i12/>. |
| --- |
| **Script number 4**  **File name:** extract_alleles.py  **Requirements**: python 2 or 3; Biopython.  **Description:** It extracts all sequences with the same length of the alleles saved in the csv file produced by script number 2 and saves them in a fasta file per sample and allele.  **How to run: python** extract_alleles_of_a_certain_length_v2.py [1] [2] [3]  [1] Coma separated text file containing genotypes. This file should contain a header containing the marker information. The next lines it contains the genotype information. Th first column should contain the sample names while the remaining the genotype information. Two columns per markers should be added allowing for heterozygote genotypes. Missing data should be coded as 0. Example:   \| samplename \| HH1_TA \| HH1_TA \| HH2_TGT \| HH2_TGT \| HH3_AAAC \| HH4_AAAC \| \| --- \| --- \| --- \| --- \| --- \| --- \| --- \| \| Sample1 \| 425 \| 425 \| 429 \| 426 \| 418 \| 418 \| \| Sample2 \| 423 \| 425 \| 429 \| 429 \| 0 \| 0 \| \| Sample3 \| 0 \| 0 \| 429 \| 429 \| 414 \| 414 \| \| Sample4 \| 427 \| 427 \| 426 \| 426 \| 418 \| 418 \|   [2] Directory containing input fastq files. These are the output from script 1.  [3] Directory to save output fasta files. These files are named in the following format: MarkerName_SampleName_Al_SequenceLength.fasta (ex: HH1_AT_Sample1_Al_425.fasta) |
| **Script number 5**  **File name:** get_consensus_and_freq.py  **Requirements**: python 2 or 3.  **Description:** It produces a consensus sequence per file keeping bases above a certain similarity threshold. For positions where this is not meet the script outputs a “N”.  **How to run:** python get_consensus_and_freq.py [1] [2] [3]  [1] Directory containing input fasta extracted based on length genotype information (output from script 4)  [2] Directory to save the consensus files. Files will be named in the following format: Marker Name_Sample Name_Al_Sequence Length_C Numer Of Sequences Used_Consensus Threashold.fasta (ex: HH1_AT_Sample1_Al_425_C881_70.fasta)  [3] Similarity of frequency threshold in an integer form. The value of 0.7 will do a 70% consensus.  **Script 6**  **File name:** correct_allele_sequence.py  **Requirements:** python 2 or 3  **Description:** In case a sequence has an ambiguous base (“N”) after the consensus and it is homozygote based on sequence length (SL), it divides the sequence into two new ones. The Ns are corrected based on the frequency that the N position shows up in the reads extracted from script 4 taking the two most frequent nucleotide combinations. In case of heterozygote genotype based on SL the sequence is not divided but only corrected with the most frequent nucleotide information.  **How to run:** python correct_allele_sequence.py [1] [2] [3] [4]  [1] fasta file containing all consensus sequences from one marker. File name should be MarkerName.fasta (ex: HH1_AT.fasta). Sequences should be named in the following format: MarkerName_SampleName_Al_SequenceLength_CNumerOfSequencesUsed_ConsensusThreashold (ex: >HH1_AT_Sample1_Al_425_C881_70)  [2] Directory containing sequences extracted based on length genotypes (output from script 4)  [3] Minimum number of sequence counts required for an allele to be condidered  [4] Name of the output fasta file |
| **Script number 7**  **File name:** call_alleles_from_fasta.py  **Requirements:** python 2 or 3.  **Description:** Uses the haplotypes obtained from the SNP correction process and converts them into allele’s numbers. If a haplotype can be assigned to more than one allele it is saved as missing data. The results are saved in tab separated text file in the format of a codominant matrix (*matrix.txt). Allele’s numbers and which haplotypes they correspond to are saved in file ending with *allelle_list.txt with the following format:  Marker 1  Allele 1: Haplotype  Allele 2: Haplotype  …  Marker 2  …  **How to run:** python call_alleles_from_fasta.py [1] [2] [3] [4] [5]  [1] Directory containing haplotypes per locus in fasta format (output from script 7)  [2] Prefix common to all input files (ex: HH in marker HH1)  [3] List of samples names to be considered. This should be a text file with one sample name per line)  [4] Prefix that should be used to save output files  [5] Minimum number of sequences required to for a haplotype to be consider |

Table S4. Amplification success shown as percentage of missing data and variability measures: Na – number of alleles, H_O_ – observed heterozygosity, H_E_ – expected heterozygosity, and PIC – polymorphism information content. Values calculated based on sequence information are represented by the superscript ^S^ while the ones based on length information by ^L^. The last seven markers separated from the remaining ones correspond to the markers excluded for the complete analyses either by excess of missing data or lack of variation.

| **Marker** | **All samples** | | | | | | | | | ***E. europaeus*** | | | | | | | | | ***E. roumanicus*** | | | | | | | | |
| --- | --- | --- | --- | --- | --- | --- | --- | --- | --- | --- | --- | --- | --- | --- | --- | --- | --- | --- | --- | --- | --- | --- | --- | --- | --- | --- | --- |
|  | **% missing** | **Na^S^** | **Na^L^** | **H_O_^S^** | **H_O_^L^** | **H_E_^S^** | **H_E_^L^** | **PIC^S^** | **PIC^L^** | **% missing** | **Na^S^** | **Na^L^** | **H_O_^S^** | **H_O_^L^** | **H_E_^S^** | **H_E_^L^** | **PIC^S^** | **PIC^L^** | **% missing** | **Na^S^** | **Na^L^** | **H_O_^S^** | **H_O_^L^** | **H_E_^S^** | **H_E_^L^** | **PIC^S^** | **PIC^L^** |
| E1 | 31,71 | 21 | 19 | 0,79 | 0,77 | 0,91 | 0,91 | 0,89 | 0,89 | 43,90 | 13 | 13 | 0,91 | 0,91 | 0,79 | 0,79 | 0,85 | 0,75 | 19,51 | 14 | 12 | 0,70 | 0,67 | 0,88 | 0,87 | 0,75 | 0,84 |
| E10 | 46,34 | 12 | 10 | 0,61 | 0,55 | 0,85 | 0,82 | 0,83 | 0,79 | 85,37 | 6 | 6 | 0,17 | 0,17 | 0,86 | 0,86 | 0,79 | 0,76 | 7,32 | 9 | 7 | 0,68 | 0,61 | 0,82 | 0,78 | 0,76 | 0,75 |
| E11 | 9,76 | 22 | 12 | 0,68 | 0,61 | 0,92 | 0,86 | 0,91 | 0,84 | 17,07 | 13 | 10 | 0,68 | 0,65 | 0,86 | 0,84 | 0,84 | 0,80 | 2,44 | 15 | 8 | 0,68 | 0,58 | 0,87 | 0,77 | 0,83 | 0,72 |
| E13 | 0,00 | 37 | 12 | 0,76 | 0,68 | 0,94 | 0,88 | 0,93 | 0,86 | 0,00 | 26 | 10 | 0,88 | 0,73 | 0,94 | 0,89 | 0,82 | 0,87 | 0,00 | 18 | 12 | 0,63 | 0,63 | 0,85 | 0,84 | 0,93 | 0,80 |
| E16 | 1,22 | 16 | 11 | 0,70 | 0,68 | 0,90 | 0,86 | 0,88 | 0,84 | 2,44 | 10 | 9 | 0,75 | 0,75 | 0,86 | 0,86 | 0,86 | 0,83 | 0,00 | 13 | 8 | 0,66 | 0,61 | 0,88 | 0,83 | 0,83 | 0,79 |
| E18 | 9,76 | 8 | 6 | 0,41 | 0,24 | 0,78 | 0,65 | 0,74 | 0,59 | 17,07 | 4 | 3 | 0,38 | 0,03 | 0,54 | 0,06 | 0,60 | 0,06 | 2,44 | 7 | 6 | 0,43 | 0,43 | 0,64 | 0,63 | 0,42 | 0,59 |
| E19 | 2,44 | 12 | 7 | 0,33 | 0,20 | 0,69 | 0,62 | 0,66 | 0,56 | 2,44 | 3 | 2 | 0,03 | 0,00 | 0,07 | 0,05 | 0,79 | 0,05 | 2,44 | 12 | 7 | 0,63 | 0,40 | 0,82 | 0,67 | 0,07 | 0,61 |
| E2 | 8,54 | 23 | 12 | 0,63 | 0,60 | 0,87 | 0,82 | 0,85 | 0,79 | 17,07 | 17 | 10 | 0,71 | 0,65 | 0,88 | 0,84 | 0,63 | 0,80 | 0,00 | 11 | 7 | 0,56 | 0,56 | 0,67 | 0,63 | 0,86 | 0,58 |
| E20 | 32,93 | 19 | 18 | 0,49 | 0,49 | 0,92 | 0,92 | 0,91 | 0,91 | 39,02 | 12 | 12 | 0,48 | 0,48 | 0,88 | 0,88 | 0,80 | 0,85 | 26,83 | 11 | 11 | 0,50 | 0,50 | 0,84 | 0,84 | 0,85 | 0,80 |
| E22 | 0,00 | 15 | 11 | 0,51 | 0,35 | 0,83 | 0,71 | 0,81 | 0,68 | 0,00 | 7 | 5 | 0,39 | 0,10 | 0,54 | 0,12 | 0,82 | 0,12 | 0,00 | 13 | 10 | 0,63 | 0,61 | 0,84 | 0,84 | 0,47 | 0,81 |
| E23 | 10,98 | 49 | 15 | 0,99 | 0,97 | 0,94 | 0,85 | 0,93 | 0,83 | 19,51 | 28 | 13 | 0,97 | 0,97 | 0,94 | 0,87 | 0,85 | 0,84 | 2,44 | 25 | 6 | 1,00 | 0,98 | 0,87 | 0,64 | 0,92 | 0,58 |
| E26 | 30,49 | 8 | 6 | 0,37 | 0,16 | 0,76 | 0,60 | 0,71 | 0,54 | 41,46 | 6 | 5 | 0,33 | 0,33 | 0,61 | 0,61 | 0,43 | 0,55 | 19,51 | 4 | 3 | 0,39 | 0,03 | 0,54 | 0,09 | 0,56 | 0,09 |
| E27 | 0,00 | 7 | 4 | 0,12 | 0,09 | 0,57 | 0,56 | 0,48 | 0,46 | 0,00 | 6 | 3 | 0,15 | 0,07 | 0,37 | 0,31 | 0,24 | 0,27 | 0,00 | 3 | 3 | 0,10 | 0,10 | 0,26 | 0,26 | 0,33 | 0,24 |
| E28 | 1,22 | 7 | 4 | 0,27 | 0,11 | 0,72 | 0,60 | 0,67 | 0,51 | 2,44 | 7 | 4 | 0,25 | 0,18 | 0,49 | 0,40 | 0,40 | 0,35 | 0,00 | 5 | 3 | 0,29 | 0,05 | 0,47 | 0,12 | 0,44 | 0,11 |
| E29 | 23,17 | 11 | 3 | 0,37 | 0,21 | 0,83 | 0,47 | 0,81 | 0,37 | 21,95 | 6 | 2 | 0,38 | 0,13 | 0,66 | 0,31 | 0,71 | 0,26 | 24,39 | 8 | 3 | 0,35 | 0,29 | 0,75 | 0,54 | 0,61 | 0,42 |
| E3 | 37,80 | 23 | 14 | 0,45 | 0,41 | 0,89 | 0,87 | 0,87 | 0,85 | 17,07 | 10 | 8 | 0,50 | 0,50 | 0,77 | 0,77 | 0,90 | 0,72 | 58,54 | 15 | 9 | 0,35 | 0,24 | 0,94 | 0,86 | 0,73 | 0,82 |
| E30 | 0,00 | 8 | 5 | 0,46 | 0,33 | 0,78 | 0,69 | 0,75 | 0,64 | 0,00 | 5 | 4 | 0,34 | 0,10 | 0,58 | 0,27 | 0,65 | 0,25 | 0,00 | 6 | 4 | 0,59 | 0,56 | 0,71 | 0,69 | 0,51 | 0,61 |
| E31 | 9,76 | 5 | 3 | 0,30 | 0,26 | 0,66 | 0,64 | 0,60 | 0,57 | 17,07 | 3 | 3 | 0,06 | 0,06 | 0,09 | 0,09 | 0,53 | 0,08 | 2,44 | 5 | 3 | 0,50 | 0,43 | 0,61 | 0,55 | 0,08 | 0,44 |
| E34 | 0,00 | 12 | 8 | 0,51 | 0,40 | 0,84 | 0,77 | 0,82 | 0,73 | 0,00 | 9 | 7 | 0,39 | 0,20 | 0,65 | 0,39 | 0,77 | 0,37 | 0,00 | 11 | 8 | 0,63 | 0,61 | 0,80 | 0,79 | 0,62 | 0,75 |
| E35 | 21,95 | 5 | 5 | 0,13 | 0,13 | 0,61 | 0,61 | 0,55 | 0,55 | 29,27 | 5 | 5 | 0,28 | 0,28 | 0,61 | 0,61 | 0,11 | 0,56 | 14,63 | 3 | 3 | 0,00 | 0,00 | 0,11 | 0,11 | 0,56 | 0,11 |
| E36 | 9,76 | 35 | 13 | 0,78 | 0,62 | 0,95 | 0,90 | 0,94 | 0,88 | 17,07 | 23 | 12 | 0,82 | 0,56 | 0,92 | 0,85 | 0,87 | 0,82 | 2,44 | 19 | 10 | 0,75 | 0,68 | 0,89 | 0,87 | 0,90 | 0,84 |
| E37 | 0,00 | 7 | 5 | 0,33 | 0,29 | 0,73 | 0,70 | 0,69 | 0,65 | 0,00 | 5 | 4 | 0,12 | 0,07 | 0,35 | 0,27 | 0,67 | 0,25 | 0,00 | 7 | 5 | 0,54 | 0,51 | 0,73 | 0,72 | 0,33 | 0,65 |
| E4 | 10,98 | 9 | 4 | 0,34 | 0,26 | 0,69 | 0,64 | 0,62 | 0,56 | 17,07 | 6 | 3 | 0,18 | 0,03 | 0,27 | 0,09 | 0,47 | 0,08 | 4,88 | 6 | 4 | 0,49 | 0,46 | 0,55 | 0,54 | 0,26 | 0,44 |
| E5 | 47,56 | 16 | 15 | 0,21 | 0,21 | 0,70 | 0,65 | 0,68 | 0,63 | 34,15 | 6 | 5 | 0,11 | 0,11 | 0,39 | 0,27 | 0,86 | 0,26 | 60,98 | 13 | 13 | 0,38 | 0,38 | 0,90 | 0,90 | 0,37 | 0,86 |
| E8 | 34,15 | 7 | 4 | 0,24 | 0,17 | 0,69 | 0,57 | 0,66 | 0,49 | 9,76 | 3 | 2 | 0,30 | 0,19 | 0,50 | 0,39 | 0,66 | 0,31 | 58,54 | 5 | 4 | 0,12 | 0,12 | 0,72 | 0,70 | 0,43 | 0,64 |
| E9 | 19,51 | 11 | 7 | 0,52 | 0,42 | 0,81 | 0,58 | 0,78 | 0,54 | 17,07 | 9 | 6 | 0,94 | 0,79 | 0,70 | 0,59 | 0,54 | 0,54 | 21,95 | 6 | 4 | 0,06 | 0,03 | 0,59 | 0,53 | 0,66 | 0,47 |
| W10 | 1,22 | 10 | 8 | 0,38 | 0,38 | 0,72 | 0,66 | 0,68 | 0,62 | 2,44 | 7 | 6 | 0,65 | 0,65 | 0,78 | 0,77 | 0,18 | 0,72 | 0,00 | 6 | 4 | 0,12 | 0,12 | 0,19 | 0,16 | 0,73 | 0,16 |
| W11 | 29,27 | 23 | 10 | 0,53 | 0,48 | 0,93 | 0,88 | 0,91 | 0,86 | 19,51 | 20 | 8 | 0,73 | 0,64 | 0,94 | 0,83 | 0,71 | 0,80 | 39,02 | 7 | 6 | 0,28 | 0,28 | 0,76 | 0,72 | 0,92 | 0,65 |
| W12 | 2,44 | 50 | 22 | 0,79 | 0,75 | 0,97 | 0,93 | 0,96 | 0,92 | 0,00 | 20 | 14 | 0,80 | 0,78 | 0,94 | 0,89 | 0,94 | 0,87 | 4,88 | 34 | 12 | 0,77 | 0,72 | 0,96 | 0,84 | 0,92 | 0,81 |
| W14 | 1,22 | 15 | 8 | 0,63 | 0,62 | 0,88 | 0,77 | 0,87 | 0,73 | 0,00 | 10 | 7 | 0,56 | 0,54 | 0,80 | 0,77 | 0,71 | 0,73 | 2,44 | 7 | 6 | 0,70 | 0,70 | 0,75 | 0,73 | 0,76 | 0,68 |
| W16 | 19,51 | 7 | 4 | 0,41 | 0,21 | 0,49 | 0,25 | 0,46 | 0,23 | 12,20 | 5 | 3 | 0,42 | 0,31 | 0,41 | 0,31 | 0,45 | 0,29 | 26,83 | 4 | 3 | 0,40 | 0,10 | 0,54 | 0,16 | 0,38 | 0,15 |
| W19 | 19,51 | 23 | 15 | 0,64 | 0,62 | 0,92 | 0,89 | 0,90 | 0,88 | 7,32 | 13 | 9 | 0,55 | 0,53 | 0,88 | 0,85 | 0,85 | 0,82 | 31,71 | 16 | 13 | 0,75 | 0,75 | 0,88 | 0,85 | 0,86 | 0,82 |
| W21 | 40,24 | 4 | 2 | 0,12 | 0,12 | 0,61 | 0,35 | 0,54 | 0,29 | 51,22 | 3 | 2 | 0,10 | 0,10 | 0,56 | 0,51 | 0,16 | 0,37 | 29,27 | 4 | 2 | 0,14 | 0,14 | 0,16 | 0,13 | 0,44 | 0,12 |
| W23 | 1,22 | 11 | 8 | 0,59 | 0,58 | 0,84 | 0,83 | 0,81 | 0,80 | 0,00 | 7 | 6 | 0,68 | 0,66 | 0,74 | 0,73 | 0,60 | 0,67 | 2,44 | 7 | 7 | 0,50 | 0,50 | 0,66 | 0,66 | 0,68 | 0,60 |
| W29 | 13,41 | 14 | 9 | 0,61 | 0,58 | 0,87 | 0,80 | 0,85 | 0,77 | 7,32 | 10 | 9 | 0,71 | 0,71 | 0,84 | 0,84 | 0,59 | 0,80 | 19,51 | 7 | 5 | 0,48 | 0,42 | 0,66 | 0,63 | 0,81 | 0,55 |
| W30 | 14,63 | 30 | 23 | 0,69 | 0,67 | 0,93 | 0,92 | 0,92 | 0,91 | 0,00 | 17 | 11 | 0,66 | 0,63 | 0,86 | 0,82 | 0,86 | 0,80 | 29,27 | 16 | 15 | 0,72 | 0,72 | 0,88 | 0,88 | 0,85 | 0,85 |
| W31 | 0,00 | 33 | 19 | 1,00 | 1,00 | 0,93 | 0,90 | 0,92 | 0,89 | 0,00 | 23 | 15 | 1,00 | 1,00 | 0,89 | 0,85 | 0,80 | 0,83 | 0,00 | 14 | 10 | 1,00 | 1,00 | 0,83 | 0,78 | 0,87 | 0,75 |
| W32 | 45,12 | 9 | 9 | 0,42 | 0,42 | 0,57 | 0,57 | 0,54 | 0,54 | 4,88 | 5 | 5 | 0,41 | 0,41 | 0,46 | 0,46 | 0,72 | 0,42 | 85,37 | 6 | 6 | 0,50 | 0,50 | 0,82 | 0,82 | 0,42 | 0,72 |
| W5 | 23,17 | 13 | 8 | 0,60 | 0,46 | 0,88 | 0,78 | 0,86 | 0,75 | 31,71 | 8 | 5 | 0,46 | 0,46 | 0,80 | 0,71 | 0,71 | 0,64 | 14,63 | 9 | 6 | 0,71 | 0,46 | 0,76 | 0,54 | 0,75 | 0,48 |
| W7 | 1,22 | 16 | 9 | 0,69 | 0,69 | 0,90 | 0,84 | 0,89 | 0,82 | 0,00 | 11 | 9 | 0,73 | 0,73 | 0,83 | 0,82 | 0,77 | 0,78 | 2,44 | 10 | 7 | 0,65 | 0,65 | 0,80 | 0,80 | 0,79 | 0,76 |
| W8 | 0,00 | 29 | 23 | 0,85 | 0,84 | 0,95 | 0,94 | 0,94 | 0,93 | 0,00 | 23 | 17 | 0,80 | 0,78 | 0,92 | 0,88 | 0,91 | 0,86 | 0,00 | 16 | 16 | 0,90 | 0,90 | 0,92 | 0,92 | 0,90 | 0,91 |
| W9 | 37,80 | 15 | 9 | 0,45 | 0,43 | 0,89 | 0,85 | 0,87 | 0,82 | 14,63 | 9 | 7 | 0,57 | 0,54 | 0,82 | 0,79 | 0,76 | 0,75 | 60,98 | 9 | 7 | 0,19 | 0,19 | 0,81 | 0,80 | 0,78 | 0,75 |
| E24 | 1,22 | 1 | 1 | 0,00 | 0,00 | 0,00 | 0,00 | 0,00 | 0,00 | 2,44 | 1 | 1 | 0,00 | 0,00 | 0,00 | 0,00 | 0,00 | 0,00 | 0,00 | 1 | 1 | 0,00 | 0,00 | 0,00 | 0,00 | 0,00 | 0,00 |
| E7 | 54,88 | 9 | 6 | 0,11 | 0,11 | 0,74 | 0,59 | 0,70 | 0,50 | 65,85 | 6 | 5 | 0,29 | 0,29 | 0,76 | 0,52 | 0,41 | 0,47 | 43,90 | 5 | 3 | 0,00 | 0,00 | 0,44 | 0,41 | 0,69 | 0,35 |
| W1 | 53,66 | 11 | 6 | 0,21 | 0,21 | 0,82 | 0,77 | 0,78 | 0,72 | 26,83 | 7 | 6 | 0,23 | 0,23 | 0,72 | 0,71 | 0,65 | 0,64 | 80,49 | 4 | 3 | 0,13 | 0,13 | 0,74 | 0,69 | 0,67 | 0,58 |
| W13 | 67,07 | 5 | 5 | 0,22 | 0,22 | 0,60 | 0,60 | 0,55 | 0,55 | 92,68 | 2 | 2 | 0,00 | 0,00 | 0,53 | 0,53 | 0,54 | 0,35 | 41,46 | 4 | 4 | 0,25 | 0,25 | 0,60 | 0,60 | 0,35 | 0,54 |
| W15 | 62,20 | 5 | 4 | 0,00 | 0,00 | 0,39 | 0,39 | 0,37 | 0,36 | 80,49 | 5 | 4 | 0,00 | 0,00 | 0,80 | 0,73 | 0,00 | 0,63 | 43,90 | 1 | 1 | 0,00 | 0,00 | 0,00 | 0,00 | 0,71 | 0,00 |
| W18 | 58,54 | 4 | 3 | 0,18 | 0,00 | 0,54 | 0,39 | 0,49 | 0,34 | 82,93 | 2 | 2 | 0,00 | 0,00 | 0,44 | 0,44 | 0,28 | 0,33 | 34,15 | 3 | 2 | 0,22 | 0,00 | 0,32 | 0,07 | 0,33 | 0,07 |
| W33 | 57,32 | 10 | 7 | 0,43 | 0,40 | 0,63 | 0,56 | 0,61 | 0,53 | 19,51 | 9 | 6 | 0,42 | 0,39 | 0,58 | 0,53 | 0,56 | 0,49 | 95,12 | 3 | 3 | 0,50 | 0,50 | 0,83 | 0,83 | 0,56 | 0,56 |


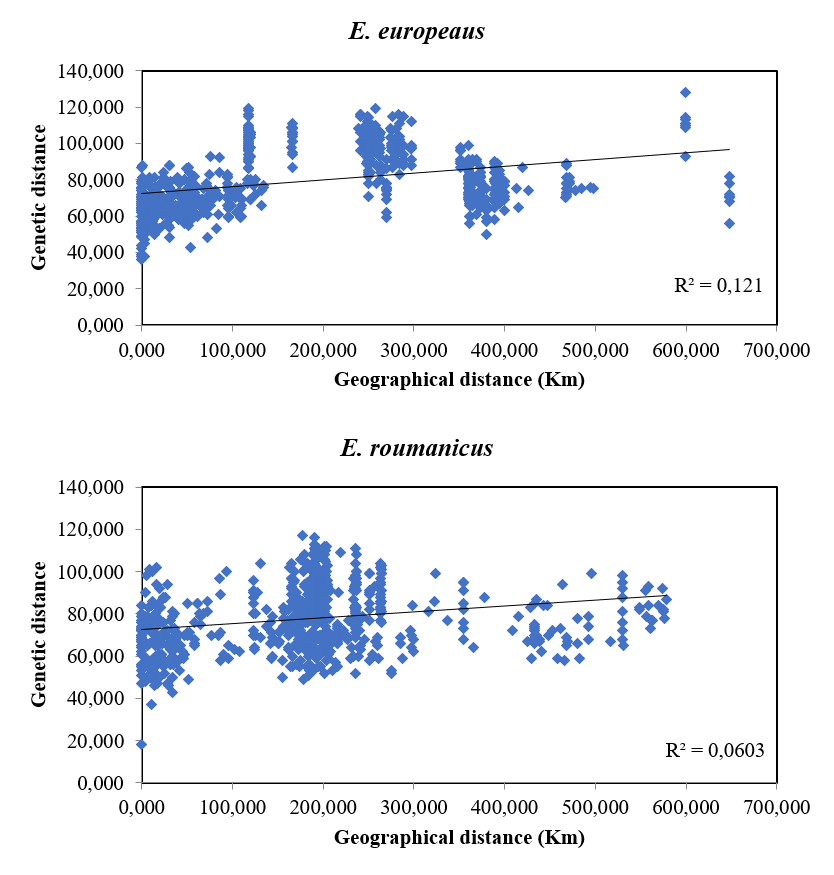


Supplementary Figure S1. Correlation between geographical and genetic distance among individuals. Distances was calculated among individuals because of lack of population sampling. Upper graph only includes individuals from E. europaeus while the bottom one from E roumanicus.

**
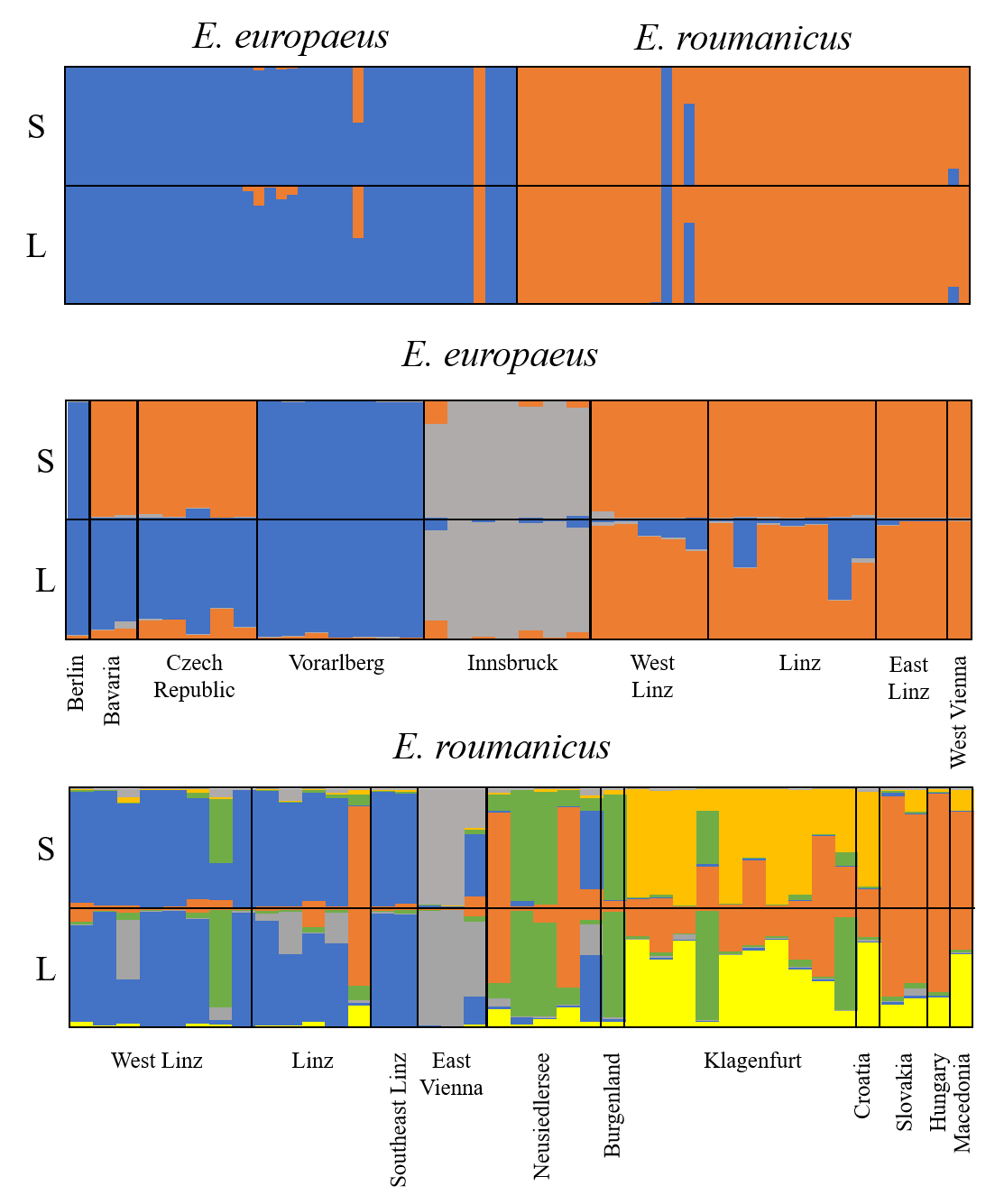
**Supplementary Figure S2. Structure analyses for all three datasets (all samples, only *E. europaeus,* only *E. roumanicus*) considering all markers and alleles called based on both sequence and length information. Only the results for the optimum K values are shown: K=2 for all samples; K=3 for *E. europaeus*; and K=5 for *E. roumanicus*.


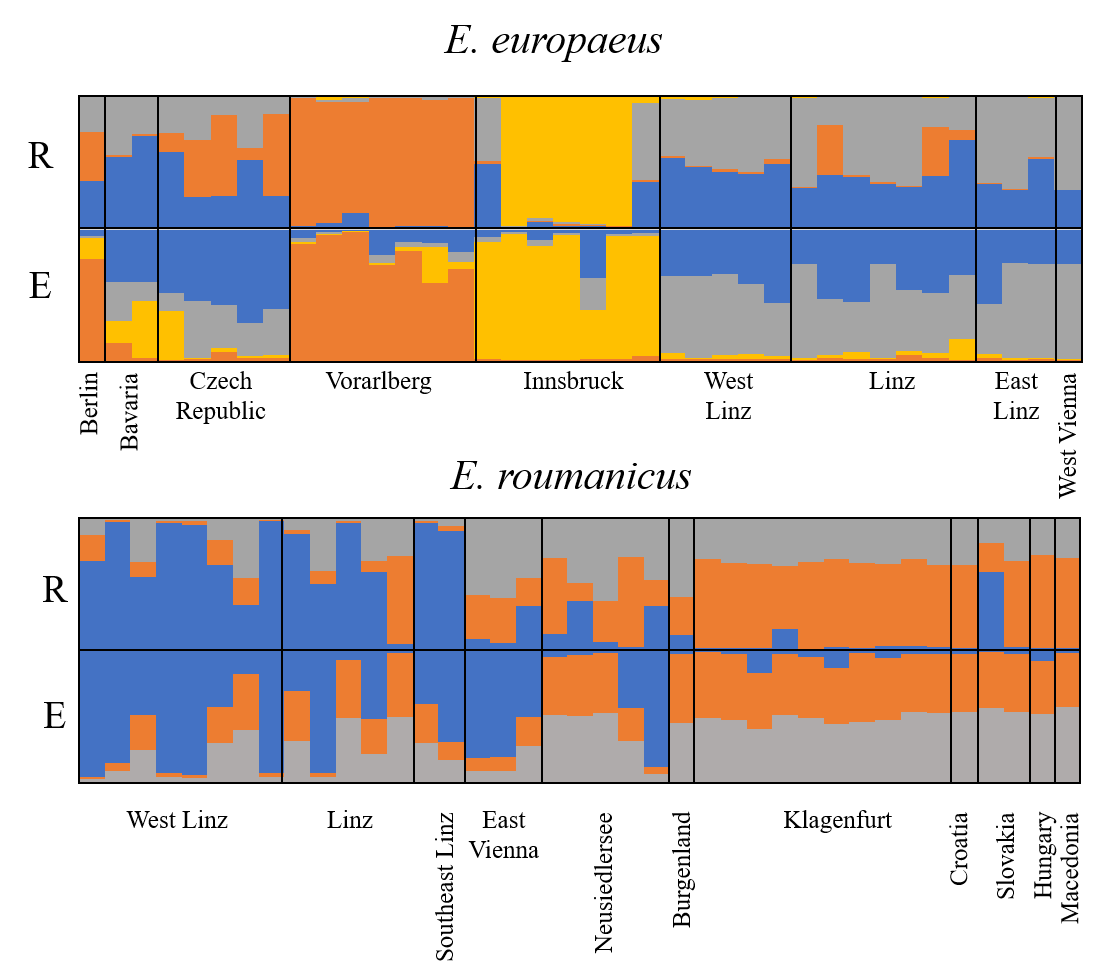
Supplementary Figure S3. Structure analyses for all three datasets (all samples, only *E. europaeus,* only *E. roumanicus*) considering markers developed specifically for *E. roumanicus* (R) and *E. europaeus* (E) and alleles called based on sequence information. Only the results for the optimum K values are shown: K=4 for *E. europaeus*; K=3 for *E. europaeus*
